# Unveiling novel features and phylogenomic assessment of indigenous *Priestia megaterium* AB-S79 using comparative genomics

**DOI:** 10.1128/spectrum.01466-24

**Published:** 2025-02-19

**Authors:** Adetomiwa Ayodele Adeniji, Chinenyenwa Fortune Chukwuneme, Emilyn Costa Conceição, Ayansina Segun Ayangbenro, Eduan Wilkinson, Elizna Maasdorp, Tulio de Oliveira, Olubukola Oluranti Babalola

**Affiliations:** 1 Centre for Epidemic Response & Innovation, School of Data Science & Computational Thinking, Stellenbosch University, Cape Town, South Africa; 2 Food Security & Safety Focus Area, Faculty of Natural & Agricultural Sciences, North-West University, Mmabatho, South Africa; 3 Department of Natural Sciences, Faculty of Applied & Computer Sciences, Vaal University of Technology, Vanderbijlpark, South Africa; 4 SAMRC Centre for Tuberculosis Research, Division of Molecular Biology & Human Genetics, Faculty of Medicine & Health Sciences, Stellenbosch University, Cape Town, South Africa; 5 SAMRC Centre for Tuberculosis Research, Division of Immunology, Faculty of Medicine & Health Sciences, Stellenbosch University, Cape Town, South Africa; 6 Department of Life Sciences, Faculty of Natural Sciences, Imperial College, Berkshire, United Kingdom; Tecnologico de Costa Rica, Cartago, Costa Rica

**Keywords:** biosynthetic gene clusters, enzymes, evolutionary genes, genome analysis, kynurenine pathway, microbial cell factories, microbial compounds, naringenin-chalcone, phosphonate

## Abstract

*Priestia megaterium* strain AB-S79 isolated from active gold mine soil previously expressed *in vitro* heavy metal resistance and has a 5.7 Mb genome useful for biotechnological exploitation. This study used web-based bioinformatic resources to analyze *P. megaterium* AB-S79 genomic relatedness, decipher its secondary metabolite biosynthetic gene clusters (BGCs), and better comprehend its taxa. Genes were highly conserved across the 14 *P*. *megaterium* genomes examined here. The pangenome reflected a total of 61,397 protein-coding genes, 59,745 homolog protein family hits, and 1,652 singleton protein family hits. There were also 7,735 protein families, including 1,653 singleton families and 6,082 homolog families. OrthoVenn3 comparison of AB-S79 protein sequences with 13 other *P. megaterium* strains, 7 other *Priestia* spp., and 6 other *Bacillus* spp. highlighted AB-S79’s unique genomic and evolutionary trait. antiSMASH identified two key transcription factor binding site regulators in AB-S79’s genome: zinc-responsive repressor (Zur) and antibiotic production activator (AbrC3), plus putative enzymes for the biosynthesis of terpenes and ranthipeptides. AB-S79 also harbors BGCs for two unique siderophores (synechobactins and schizokinens), phosphonate, dienelactone hydrolase family protein, and phenazine biosynthesis protein (phzF), which is significant for this study. Phosphonate particularly showed specificity for the *P. megaterium* sp. validating the effect of gene family expansion and contraction. *P. megaterium* AB-S79 looks to be a viable source for value-added compounds. Thus, this study contributes to the theoretical framework for the systematic metabolic and genetic exploitation of the *P. megaterium* sp., particularly the value-yielding strains.

**IMPORTANCE:**

This study explores microbial natural product discovery using genome mining, focusing on *Priestia megaterium*. Key findings highlight the potential of *P. megaterium*, particularly strain AB-S79, for biotechnological applications. The research shows a limited output of *P. megaterium* genome sequences from Africa, emphasizing the importance of the native strain AB-S79. Additionally, the study underlines the strain’s diverse metabolic capabilities, reinforcing its suitability as a model for microbial cell factories and its foundational role in future biotechnological exploitation.

## INTRODUCTION

One viable way to revitalize the natural product discovery pipeline is through microbial natural product discovery research and genome mining technology (1). By leveraging the diversity of microbial metabolites and the capacity of genomics, researchers can discover new biomolecules (e.g., enzymes, vitamins, antibiotics) urgently needed in public health to combat resistant pathogens and minimize dependence on synthetic compounds (2).

Alongside other model organisms like *Escherichia coli, Streptomyces lividans,* and *Bacillus subtilis*, *Priestia megaterium* makes an appealing microbial cell factory, owing to its impressive genome (spanning 4 Mb–7 Mb), several patents, and industrial applications (3, 4). Although *E. coli* is still the dominant cell-free system used in recombinant enzyme expression (5), various new cell-free systems from other key prokaryotic expression systems, including *P. megaterium*, *Vibrio natriegens*, *Pseudomonas putida*, *B. subtilis*, *Clostridium autoethanogenum*, and *Streptomyces* spp., are becoming readily available (6–8). *P. megaterium*, a gram-positive endospore-forming rod formerly known as *Bacillus megaterium* (9), and often called *Priestia* “big beast” (“megaterium” translates “big beast”), has at least 100 times the volume of *E. coli* (10). *E. coli* cells grow to ~0.5 µm^3^ (0.5 × 0.5 × 2), while *P. megaterium* cells can grow to over 60 µm^3^ (2.5 × 2.5 × 10) (10, 11). *P. megaterium* offers distinct advantages over *E. coli*, particularly for applications requiring complicated, endotoxin-free, safer protein secretion, and more scalable manufacturing methods (4, 8, 10).

*P. megaterium* is used in biotechnological applications for producing cobalamin (vitamin B12), enzymes, pigments, polymers, recombinant proteins, and other vitamins (11–13). It is recognized for its high protein secretion capacity, easy cultivation on valuable carbon sources, an array of commercially accessible expression vectors for generating unconventional recombinant proteins at the grams per liter magnitude, and ability for whole-cell transformation (4, 10, 14). *P. megaterium*-derived recombinant plasmids have renowned stability even in the absence of selective antibiotics (4, 10, 15), and several of its genomes encode or are used in producing cytochrome P450 enzymes, known to catalyze challenging chemical reactions (4, 14, 16). Using green fluorescent protein (GFP) monitoring, *P. megaterium* (134 ng µL−1 GFP) outperforms other prominent gram-positive hosts such as *S. lividans* 66 (~100 –400 ng µL−1) and *B. subtilis* (21.6 ng µL−1) regarding protein yield (7). *P. megaterium* expresses numerous genes required for natural competence formation (4), putting it on par with other microbial cell factories (14).

*P. megaterium* is metabolically versatile, being able to utilize various carbon sources and colonize a variety of environments, including wine, honey, fish, raw meats, seawater, human oral cavity, and plant endophytic zones (17–19). Its survival in extreme environments such as mine tailings (20), its applications in plant growth promotion, and bioremediation of heavy metal-contaminated environments have been documented (21–23). The ability of diverse microbial species (bacteria, fungi, and viruses) to produce secondary metabolites with significant biotechnological applications is widely documented (24, 25). However, the difficulty in identifying the genes that regulate the biosynthesis of these metabolites before chemical characterization frequently stymies discovery. With the emergence of genome-based approaches, it is becoming easier to identify and characterize gene clusters in microbial genomes (2), enabling the *in vitro* characterization of various novel bioactive compounds such as mupirocin, fengycin, doxorubicin and epothilone in several microbial genera such as *Streptomyces*, *Pseudomonas*, *Bacillus*, and Actinomycetes (26–28).

Given that secondary metabolite gene clusters often exhibit species specificity within closely related bacteria (29), this study explores *P. megaterium* AB-S79’s genome in search of valuable biomolecules using web-based bioinformatic tools. Web-based bioinformatic platforms have become indispensable repositories and tools for multi-omics investigations (30–32), particularly in low- and middle-income countries where the cost of multi-omics and bioinformatics integration is still a huge burden. Since its reclassification, this is the earliest pangenome-scale comparative investigation on *P. megaterium* species. Furthermore, since the previously sequenced 5.7 Mb genome of the native *P. megaterium* AB-S79 (33) lacked comprehensive annotation, comparative analysis, and phylogenetic placement, we analyzed the genome to showcase the unique attributes of the strain. We also describe AB-S79’s evolutionary history through phylogenomic and phylogeographic approach. Considering *P. megaterium* sp. biotechnological potential, we further ascertained the global distribution of its sequenced genomes to identify their sources. Lastly, we compared AB-S79’s biosynthetic capacity with other closely related *Priestia* and distantly related *Bacillus* species.

## MATERIALS AND METHODS

### *P. megaterium* AB-S79 subsystem analysis

**Fig 1:**
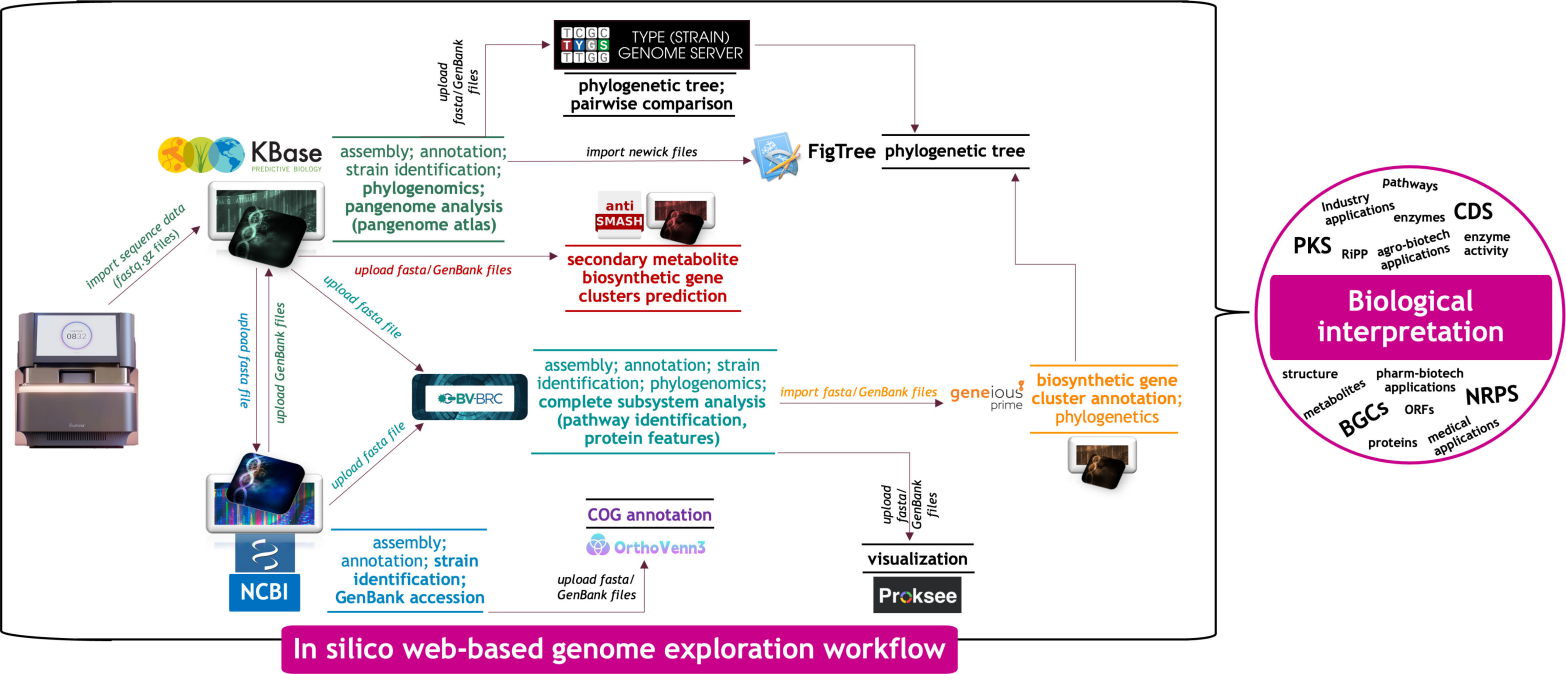
Graphical illustration of the web-based tools used in the exploration of *P. megaterium* AB-S79 genome.

The graphical workflow for the *in silico* web-based exploration of the *P. megaterium* AB-S79 genome is illustrated in Fig. 1. The isolation of *P. megaterium* AB-S79, its identification, and metal resistance analysis were previously described by Ayangbenro (34). Following, Adeniji et al. (33) reported its genomic DNA isolation, draft genome sequencing, and assembly. The draft whole-genome shotgun project was deposited in DDBJ/ENA/GenBank under the accession number JAUCND000000000.1. A complete subsystem analysis of the *P. megaterium* AB-S79 genome was conducted using the online Bacterial and Viral Bioinformatics Resource Center (BV-BRC: https://www.bv-brc.org/; v.3.35.5) (35). This analysis involved identifying proteins responsible for executing specific biological processes or forming structural complexes in the AB-S79 genome. These proteins encompassed genes showing similarity to known transporters, virulence factors, and drug target genes. The subsystem circular view was generated, and the specialized genes were identified. The antimicrobial resistance (AMR) genes in *P. megaterium* AB-S79 was further identified using BV-BRC Genome Annotation Service that employs a k-mer-based AMR gene detection method that makes use of the BV-BRC’s curated collection of representative AMR gene sequence variants and assigns the respective AMR gene functional annotation, broad mechanism of AMR, drug class, and, in some cases, specific antibiotic resistance.

### Comparative genomic analysis and genomic metrics

Considering best-match type strain for *P. megaterium* AB-S79 on the National Center for Biotechnology Information (NCBI) server (https://www.ncbi.nlm.nih.gov/datasets/genome/GCF_001591525.1/), 13 highly similar genomes (GenBank complete sequences producing significant alignments) including the reference strains *P. megaterium* NBRC 15308 = ATCC 14581 (GCA_001591525.1) and *P. megaterium* DSM32 (GCA_029536995.1) were retrieved from the NCBI database on 5 September 2023, using the blast suite download tool. The 13 retrieved genomes and AB-S79 genome were merged afterward and called “query-genomes14” for use in subsequent analyses (Table 1). Unless otherwise noted, all software used the default mode settings.

**TABLE 1:** Comparative summary of the “query-genomes14” (*Priestia megaterium* AB-S79 genome and 13 NCBI genomes)

Genome (accession)	Check M complete	Genome size (bp)	N50	G + C%	CDS^*a*^	tRNA	5S rRNA	16S rRNA
*P*. *megaterium* WSH-002 (GCF_000225265.1)	99.41%	4,983,975	4,983,975	38.24%	5,180	99	10	10
*P*. *megaterium* NCTC10342 (GCA_900445485.1)	98.28%	4,542,023	4,542,023	38.08%	4,516	118	13	12
*P*. *megaterium* Q3 (GCF_001050455.1)	99.43%	5,153,539	5,153,539	38.26%	5,204	113	13	13
*P*. *megaterium* B-12 (GCA_030270625.1)	99.43%	5,127,551	5,127,551	38.33%	5,223	115	12	12
*Priestia megaterium* DSM319 (GCA_029537015.1)	99.14%	5,102,745	5,102,745	38.15%	5,177	118	12	12
*P*. *megaterium* NBRC15308 (GCA_001591525.1)	98.25%	5,302,122	4,974,737	38.35%	5,450	121	16	15
*P*. *megaterium* DSM_2894 (GCA_029857455.1)	99.43%	4,974,737	4,974,737	38.31%	5,110	118	14	13
*P*. *megaterium* DSM32 (GCA_029536995.1)	99.43%	5,294,407	5,294,407	38.09%	5,370	123	14	13
*P*. *megaterium* IN103 (GCA_008728535.1)	98.12%	5,074,523	5,074,523	38.36%	5,141	114	13	13
*P*. *megaterium* KNU-01 (GCA_024169105.1)	100.0%	10,120,194	5,145,457	38.28%	10,368	249	30	27
*P*. *megaterium* PHP1706 (GCA_030548605.1)	100.0%	10,034,199	5,059,462	38.27%	10,252	230	27	26
*P*. *megaterium* SRCM125040 (GCA_028548275.1)	100.0%	10,067,538	5,092,801	38.31%	10,209	245	29	26
*P*. *megaterium* TG1-E1 (GCA_003074515.1)	98.25%	5,132,630	4,974,737	38.38%	5,259	134	18	18
*P*. *megaterium* AB-S79 (GCA_030372795.1)	99.43%	5,676,272	840,958	37.5%	6,198	56	2	2

^*a*^ CDS, Coding sequences.

A genome set was built on the United States Department of Energy Systems Biology Knowledgebase (Kbase: http://kbase.us/) (36), narrative interface using the Build Genome Set app (v.1.7.6). The Compute Pangenome software v.0.0.7 was used to compute a unified pangenome group for the “query-genomes14.” The Kbase Compare Genomes from the Pangenome software v.0.0.7 was used for evaluating and summarizing isofunctional and homologous gene families in the pangenome. Gene overlaps (gene correlations) in the “query-genomes14” viewed using the Pangenome Circle Plot software v.1.2.0. Throughout the analyses, the *P. megaterium* AB-S79 genome served as the base genome for ordering the ortholog clusters in the query genomes.

### Phylogenomic analysis

For the phylogenomic analyses, we used Kbase’s Insert Genome into Species Tree software v.2.2.0 which allows users to construct a species tree using a subset of 49 core universal genes defined by COG (Clusters of Orthologous Groups) gene families. Briefly, the “query-genomes14” (Table 1) was combined with sets of closely related genomes selected from the public Kbase genomes function (import of RefSeq). Next, in Kbase, the “query-genomes14” along with the other selected Kbase genomes undergo trimming, multiple sequence alignment (MSA for each COG family), and concatenation. Thereafter, a phylogenetic tree was reconstructed using the default settings of FastTree2 (v.2.1.11) (37). Additionally, the fasta file of *P. megaterium* AB-S79 genome was uploaded on the Type Strain Genome Server for a Genome BLAST Distance Phylogeny (GBDP) analysis and pairwise comparison (https://tygs.dsmz.de/) (38). The resulting phylogenetic trees were compared with the phylogenetic tree generated in the BV-BRC comprehensive genome analysis report.

To determine the phylogeographic positioning of the *P. megaterium* AB-S79 isolate, a metadata inquiry for the *P. megaterium* species was done on the BV-BRC database to acquire the total *P. megaterium* taxa (genome) representatives by geographic distribution [https://www.bv-brc.org/search/?and(keyword(Priestia),keyword(megaterium))]. Genome duplicates, false, deprecated, and low-quality genomes were filtered out to extract the representative BV-BRC genomes for the corresponding NCBI taxon. Phylogenetic trees in BV-BRC were rendered using Archaeopteryx.js (https://www.bv-brc.org/docs/quick_references/services/archaeopteryx.html), and the trees were obtained by extracting subtrees from the global phylogenetic tree of bacteria provided by the Genome Taxonomy Database (GTDB) project (https://gtdb.ecogenomic.org) (39). Because the number of representative genomes identified by the BV-BRC-GTDB approach for a given taxon is frequently too large for convenient display, an upper limit is imposed to filter out genomes, typically limiting visualizable genomes to their immediate ancestral node (https://www.bv-brc.org/docs/quick_references/services/archaeopteryx.html). This resulted in trees with fewer nearly identical tips and a better representation of diversity. The Phylogeny Tab and Phylogenetic Tree Viewer on the BV-BRC web server were then launched (https://www.bv-brc.org/view/Taxonomy/1404-view_tab=phylogeny) for visualization of the *P. megaterium* taxon (reference strains) level tree.

### Biosynthetic gene cluster, gene, and pathway analysis

OrthoVenn3 (OrthoMCL clustering algorithm: default settings) (https://orthovenn3.bioinfotoolkits.net/home; [40]) was also used to annotate the COG of proteins to further highlight the uniqueness of *P. megaterium* AB-S79 in comparison to other *P. megaterium* strains, other *Priestia* spp., and six other distantly related *Bacillus* species. Protein sequence data (fasta format) of two subsets of the “query-genomes14” (Subset_A and Subset_B), seven other *Priestia* spp. (*Priestia aryabhattai* K13 [GCA_002688605.1]; *Priestia flexa* DMP08 [GCA_021441905.1]; *Priestia filamentosa* DSM 27955 [GCA_002237735.1]; *Priestia endophytica* DSM 13796 [GCA_900115845.1]; *Priestia taiwanensis* CGMCC 1.12698 [GCA_014638355.1]; *Priestia abyssalis* DSM 25875 [GCA_002019595.1]; *Priestia veravalensis* SGD-V-76 [GCA_001457055.1]), and six other *Bacillus* spp. (*Bacillus subtilis* subsp. subtilis str. 168 [GCA_000009045.1]; *Bacillus velezensis* FZB42 [GCA_000015785.2]; *Bacillus licheniformis* ATCC 14580 [GCA_034478925.1]; *Bacillus amyloliquefaciens* GKT04 [GCA_019396925.1]; *Bacillus thuringiensis* serovar berliner ATCC 10792 [GCA_000161615.1]; *Bacillus cereus* TG1-6 [GCA_003013315.1]) were downloaded from the NCBI server for the OrthoVenn3 analysis.

To elucidate the biosynthetic gene clusters (BGCs), genes, and pathways in the *P. megaterium* AB-S79 genome, antiSMASH (v.7.1.0) (41), Geneious Prime, and BV-BRC tools were integrated. The antiSMASH profile of AB-S79 was also compared with profiles of the other “query-genomes14” strains, seven *Priestia* spp., and six *Bacillus* species. A GenBank-formatted nucleotide file of the genomes was uploaded and submitted on the antiSMASH bacterial site for rapid genome-wide analysis of secondary metabolite BGCs in the AB-S79 genome. To annotate potentially known BGC, the antiSMASH interfaces with the Minimum Information about a Biosynthetic Gene (MIBiG) cluster site—MIBiG serves as a reference to highlight the possible similarity between the query BGC and the known BGC database. The AB-S79 GenBank file was also imported into Geneious Prime (v.2024.0.7) for manual BGC identification, and thereafter, a comprehensive pathway-subsystem analysis was conducted on the BV-BRC server.

### Data analysis and visualization

Except where notified, all retrieved data were analyzed and visualized in Excel v.16.83 and Numbers v.13.2 software. Circular atlas was generated by Proksee (42). Three-dimensional structures/models of proteins predicted by Geneious Prime were generated by homology inference on the Uniprot web server (https://www.uniprot.org/) (43).

## RESULTS

### Complete subsystem analysis of the AB-S79 genome

**Fig 2:**
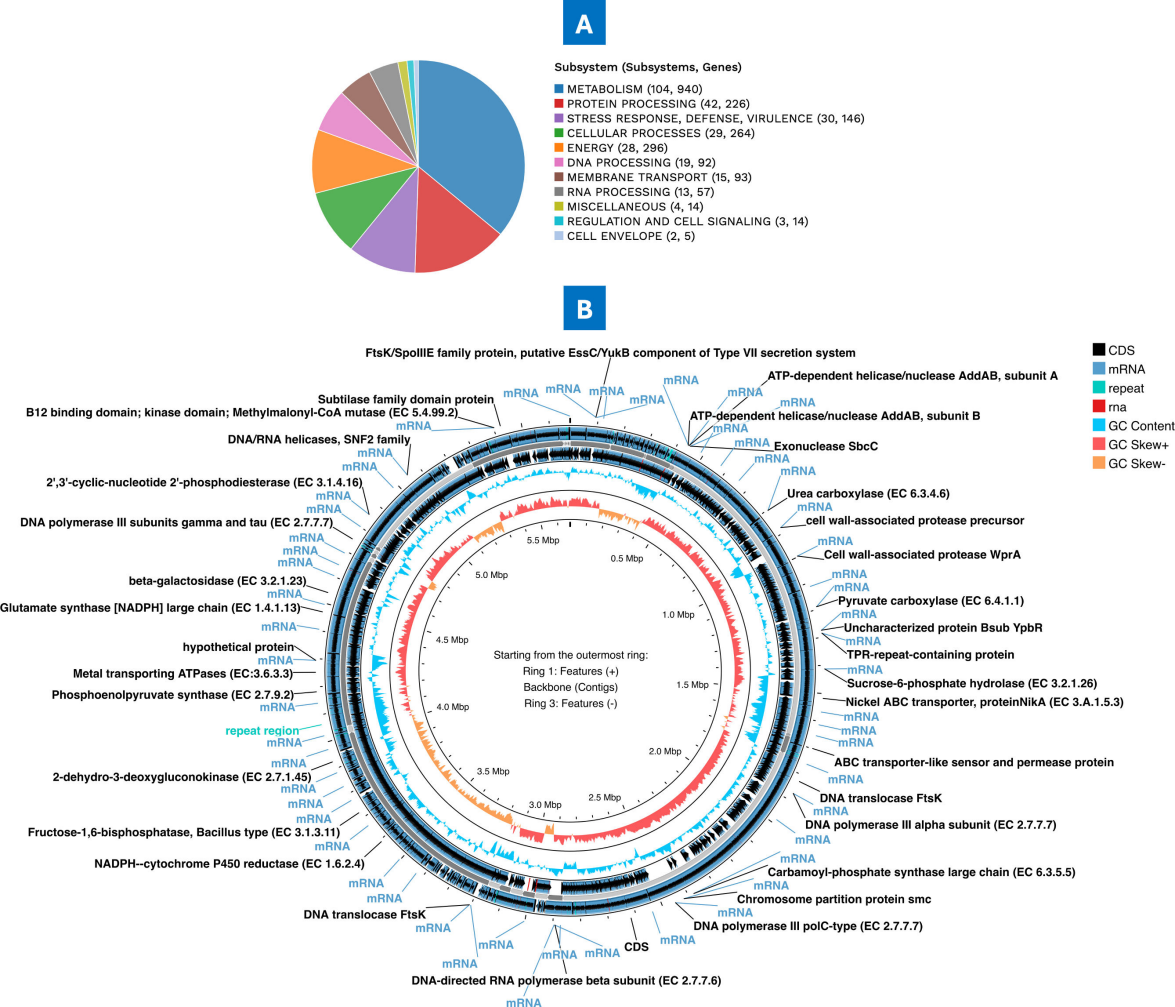
(**A**). Subsystem overview of *Priestia megaterium* AB-S79 genome; (**B**) circular atlas of *Priestia megaterium* AB-S79; innermost ring (GC skew + rRNA), inner ring (GC content), outer rings (mRNA +CDS).

The complete subsystem annotation of *P. megaterium* AB-S79 revealed that its genome has hypothetical proteins, proteins with functional assignments, and cross-genus protein families (PATRIC cross-genus family [PGFams]; specific to the BV-BRC’s annotation) (Table 2). The subsystem overview of *P. megaterium* AB-S79 and circular atlas were generated by BV-BRC and Proksee (Fig. 2A and B). In the subsystem overview (Fig. 2A), AB-S79’s genome boasts of 104 and 940 metabolism subsystems and genes, respectively; 29 subsystems and 264 attributes were allocated to cellular processes. AB-S79 genome has 42 subsystems and 226 genes specific for protein processing (Fig. 2A). From the outer to the inner rings, the circular map displays the following: contigs, CDS on both the forward and reverse strands, mRNA genes, RNA, GC content, and GC skew (Fig. 2B). The subsystem to which these genes belong is represented by the colors of the CDS on the forward and reverse strands. The total count of specialized genes plus the specific repository database where homology was discovered are displayed in Table 3. In Table 3, genes homologous to known drug targets, transporters, virulence factors, and antibiotic resistance genes were also highlighted. Table S1 lists the AMR genes identified in this genome along with the associated AMR mechanism.

**TABLE 2:** Protein feature profile of *Priestia megaterium* AB-S79^*a*^

Annotation features	Feature statistics/counts
Hypothetical proteins	2,070
Proteins with functional assignments	4,126
Proteins with EC number assignments	1,236
Proteins with GO assignments	1,038
Proteins with Pathway assignments	945
Proteins with PGfam assignments	5,878

^*a*^ EC, enzyme commission; GO, Gene Ontology; PGfam, PATRIC cross-genus family.

**TABLE 3:** Specialty genes profile of *Priestia megaterium* AB-S79 genome^*a*^

Specialty genes	Source	Genes
Antibiotic resistance	CARD; NDARO; PATRIC	2; 1; 47
Drug target	DrugBank	20
Transporter	TCDB	28
Virulence factor	PATRIC_VF; VFDB; Victors	3; 1; 6

^*a*^ CARD, Comprehensive Antibiotic Resistance Database; NDARO, National Database of Antibiotic Resistant Organisms; PATRIC, PathoSystems Resource Integration Center; TCDB, Transporter Classification Database; VFDB, virulence factor data.

### Compute pangenome, compare genomes in pangenome, and phylogenomics

**Fig 3:**
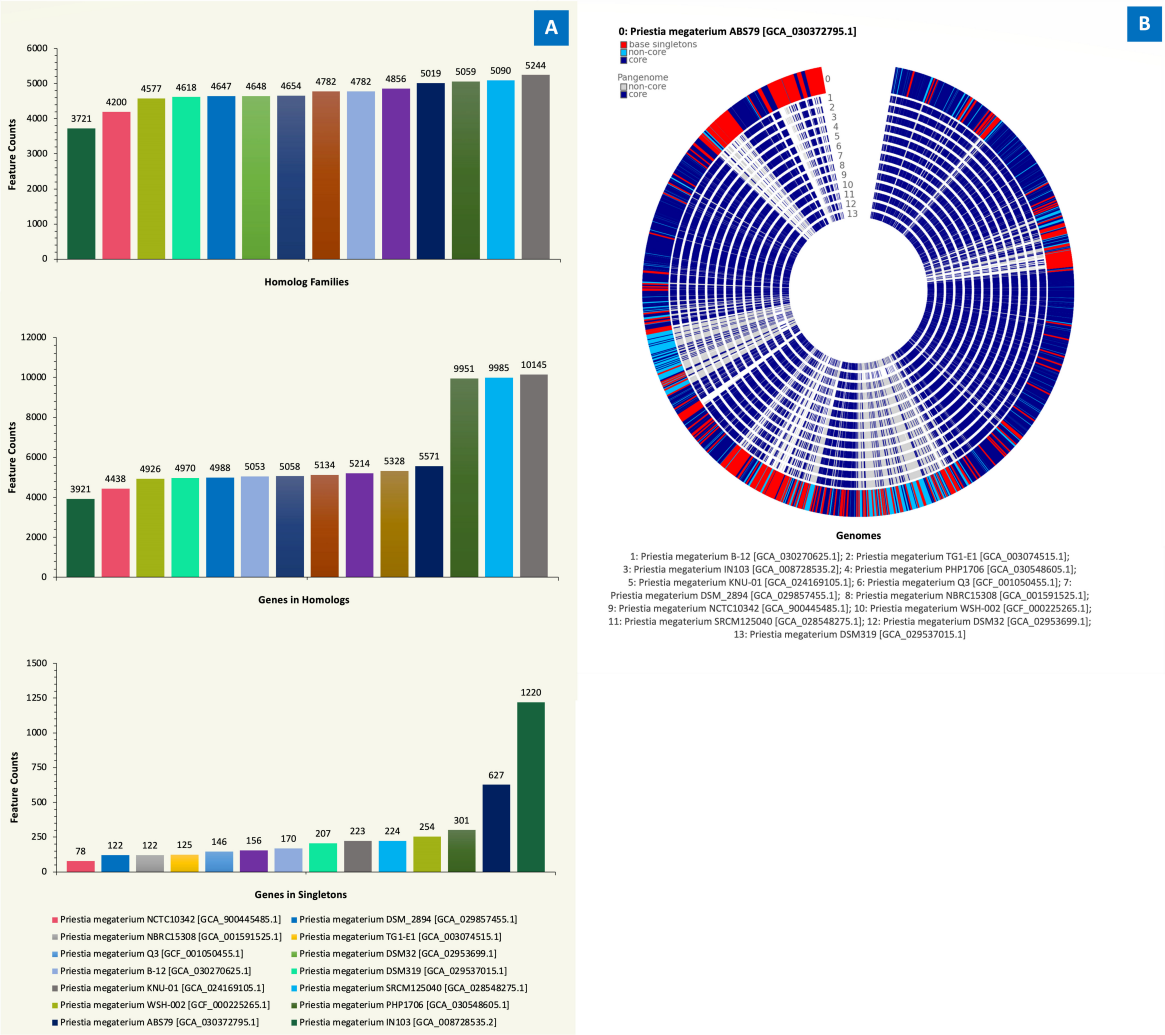
Full distribution of the “query-genomes14” pangenome attributes and their corresponding pangenome circle plot. (**A**) Illustrates the homolog families, genes in homologs, and genes in singletons; (**B**) highlights the overlaps for the “query-genomes14” (genes attributed to AB-S79 genome are in red and blue).

The computed pangenome by Kbase revealed the putative protein-coding genes (Pcgs) and core protein families within the pangenome of “query-genomes14.” Overall, from the “query-genomes14” analysis, a total of 61,397 Pcgs are involved with translation. From the 61,397 Pcgs, 59,745 are in the homolog families (Hfs), while 1,652 are in singleton families (Sfs). A total of 7,735 protein families were identified, 6,082 associated with Hfs and 1,653 Sfs. The complete distribution of the pangenome attributes (homolog families, genes in homologs, and genes in singletons) for the “query-genomes14,” and their corresponding pangenome circle plot is shown in Fig. 3A and B. A comparative summary of the genomic attributes of the “query-genomes14” was generated (Table 1).

**Fig 4:**
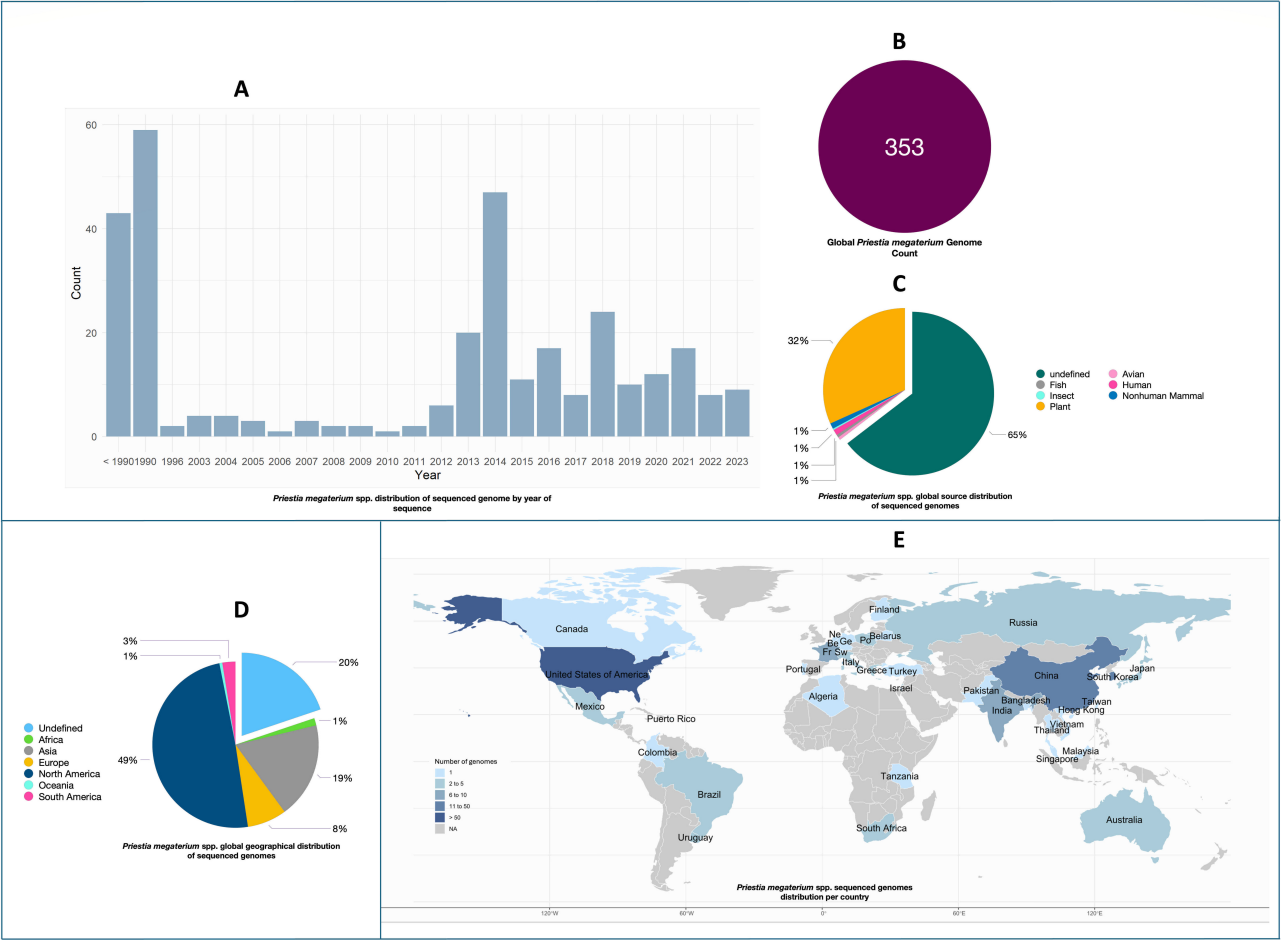
Illustrates the global *P. megaterium* taxa distribution. (**A**) *P. megaterium* distribution by sequencing year; (**B**) total *P. megaterium* sequenced genome count; (**C**) *P. megaterium* sequenced genome count by sampling sources; (**D and E**) *P. megaterium* taxa sequenced genome count by geographic locations.

**Fig 5:**
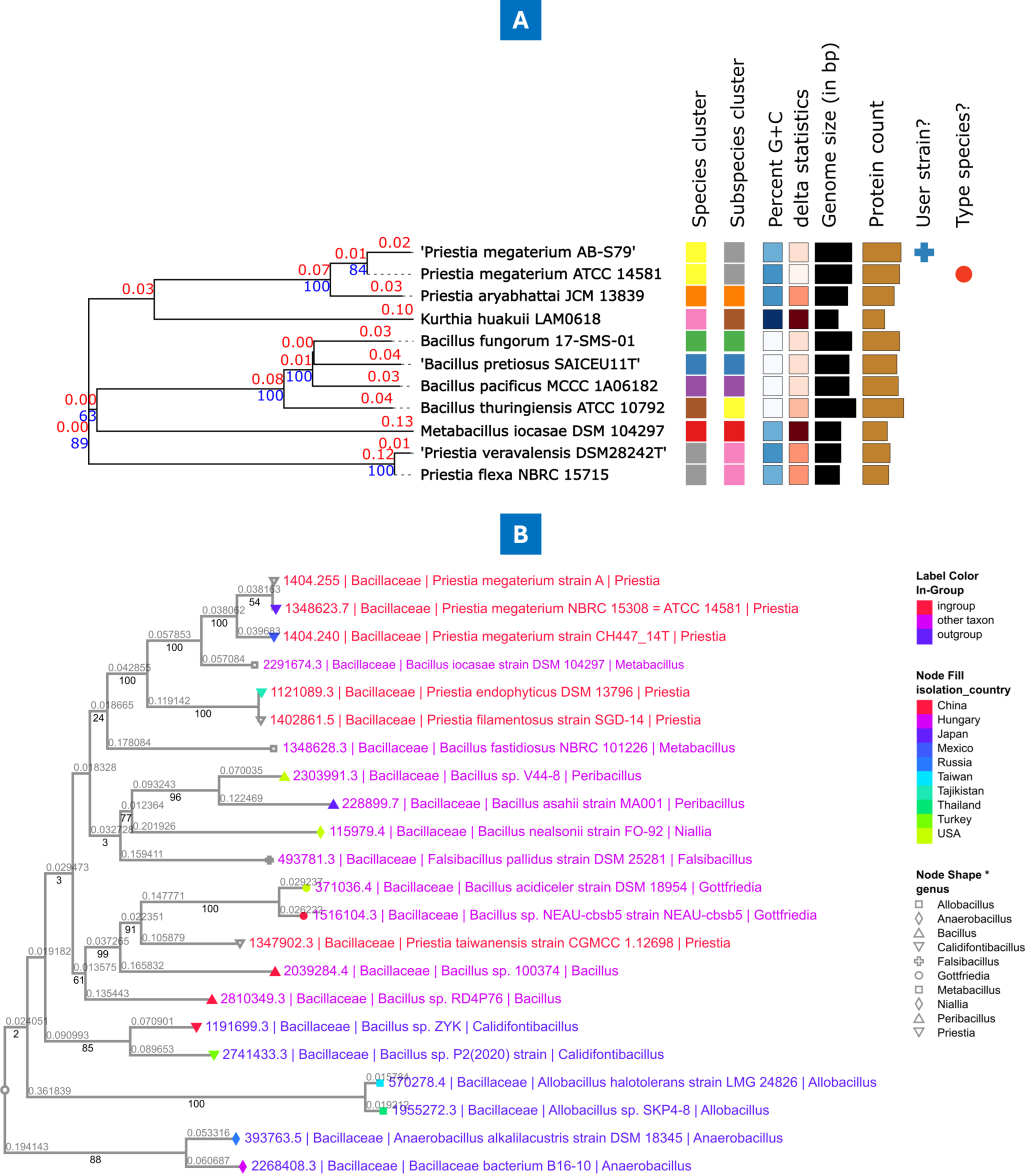
(**A**) *P. megaterium* AB-S79 phylogeny tree inferred with FastME 2.1.6.1 on the GBDP server based on GBDP distances calculated from genome sequences (38, 44). The GBDP distance formula, d5, serves to scale the branch lengths. Figures above branches are GBDP pseudo-bootstrap support values >60% from 100 replications, with a mean branch support of 65.6% (38). A midpoint rooting was done on the tree; (**B**) GTDB reference phylogeographic tree for the *P. megaterium* taxon.

The species tree of the “query-genomes14” based on protein sequence analysis showed *P. megaterium* AB-S79 clustering closely with *P. megaterium* DSM 319 (Fig. S1A and B). For geographic distribution and positioning, by collection year, 1990 had the highest value of *P. megaterium* sequenced (59 [17%]) followed by the year 2014 (47 [13%]); 38 (11%) of the sequenced genomes have no year of collection information (Fig. 4A). Total global *P. megaterium* sp. genome sequenced after duplicate, false, deprecated, and poor genomes were filtered out from the BV-BRC metadata was 353 (Fig. 4B). Comparing host groups, genomes sequenced from plant isolates had the largest percentage (112 [32%]) (Fig. 4C). North America (174 [49%]) and the United States of America (171 [48%]) had the highest count of *P. megaterium* sequenced, respectively, in terms of isolation country and geographic group (Fig. 4D and E). Africa had only five genomes sequenced, three were from South Africa including *P. megaterium* AB-S79. Figure 5A and B shows the GBDP *P. megaterium* AB-S79 phylogenetic tree and the GTDB reference phylogeographic tree for the *P. megaterium* taxon, respectively, with AB-S79 clustering closely with the reference *P. megaterium* NBRC 15308 = ATCC 14581 (purple node). With the GTDB clustering, China, Japan, and Mexico were the isolation countries of the *P. megaterium* reference genomes (Fig. 5B), indicating the locations where the species has been studied extensively.

### *P*. *megaterium* AB-S79 genome biosynthetic gene clusters, genes, and pathways

#### OrthoVenn3 analysis

**Fig 6:**
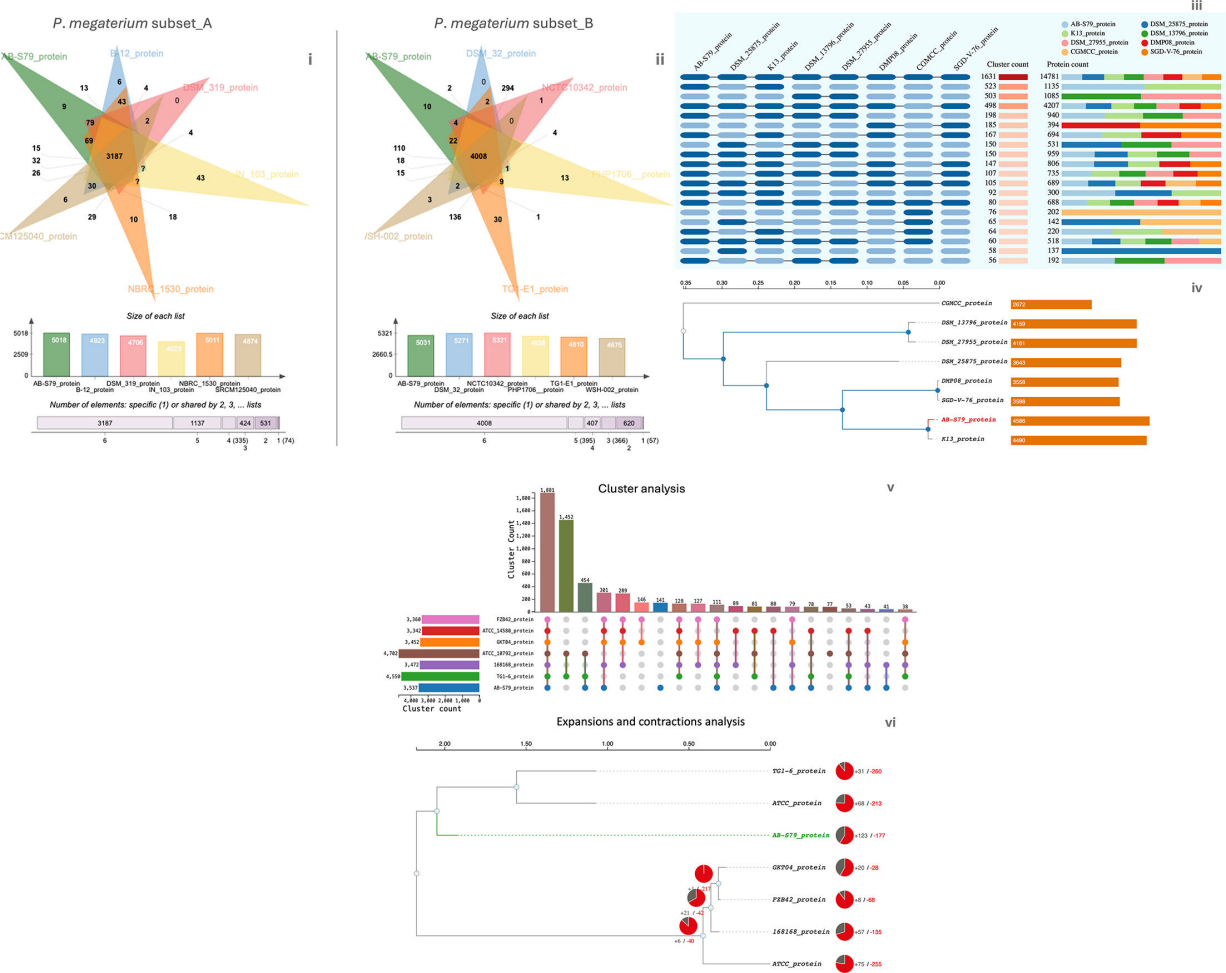
Highlight of orthologous gene clusters across the “query-genomes14” subsets (Subset_A and Subset_B; representing AB-S79 and other closely related *P. megaterium* strains) based on OrthoVenn3.(i). AB-S79 with Subset_A of “query-genomes14”; (ii). AB-S79 with Subset_B of “query-genomes14.” The Venn diagram depicts the unique and shared orthologous gene clusters, complemented by a bar chart that quantitatively details the number of clusters for each strain. The symbol “?” signifies anonymous proteins that do not fall into the orthologous groupings. Proteome comparison of *Priestia megaterium* AB-S79 orthologous gene clusters (COG) with other closely related *Priestia* spp. based on OrthoVenn3. (iii) Occurrence plot shows the number of orthologous clusters in each spp., as well as the number of unique and shared homologous gene clusters among species. (iv) Phylogenetic tree describing the evolutionary timeline and relationship between *P. megaterium* AB-S79 and other closely related *Priestia* spp. based on the identification of highly conserved single-copy genes. Genome size of each strain is written in the bar charts. Proteome comparison of strain AB-S79 and distantly related type strains of the *Bacillus* genus based on OrthoVenn3. (v) UpSet plot highlights the unique and shared orthologous clusters among species. The left horizontal bar chart depicts the number of orthologous clusters per spp., while the right vertical bar chart illustrates the number of orthologous clusters shared among the species. (vi) Shows the evolution of gene families and differences between species; pie charts detail the expansion (gray) and contraction (red).

Comparative COGs prediction by OrthoVenn3 on protein sequences of (i) two “query-genomes14” subsets, (ii) seven *Priestia* spp., and (iii) six *Bacillus* spp. generated interesting results. Subset_A of the *P. megaterium* “query-genomes14” formed 5,688 clusters, 59 overlaps (when one or more members of a cluster are shared by different clusters), 2,799 single-copy clusters (single-copy genes in each strain species), 31,827 proteins, and 872 singletons (2.74%) (without orthologs) between the genomes. From these, 3,187 clusters and 20,381 proteins were shared among Subset_A genomes (Fig. 6i). AB-S79 had the highest protein and cluster count (5,806; 5,018) in the overall protein and cluster count (31,827; 5,688) (Table 4). AB-S79 also had the highest abundance (3,488; 17.11%) in Subset_A (Table 4). Subset_B formed 5,853 clusters (2.48% singletons), 52 overlaps, 3,552 single-copy, and 33,246 proteins between the strains (Fig. 6ii). From these, 4,008 clusters and 25,528 proteins were shared among Subset_B genomes (Fig. 6i). Notably, AB-S79 had the most shared protein (absolute [4,359]; relative [17.08%]) abundance (Table 5). In Subset_A (Fig. S2i), AB-S79’s closest relative was DSM_319_protein correlating the other phylogenetic results. Besides AB-S79’s having the highest expansion (+104) and lowest contraction (−47), the *P. megaterium* strains gene family variation exhibited more contractions (Fig. S2ii). DSM_319_protein had the lowest expansion (+4), while SRCM125040_protein had the highest contraction (−262). In Fig. S2iii and iv, AB-S79 had distinct clustering though with the highest expansion.

**TABLE 4:** Summary of *Priestia megaterium* AB-S79 orthologous gene clusters (COG) relative to closely related *Priestia megaterium* strains as per OrthoVenn3

*Priestia megaterium* strains	Proteins	Clusters	Singletons	Protein absolute abundance	Protein relative abundance
Subset_ A					
AB-S79_protein	5,806	5,018	201	3,488	17.11%
NBRC_1530_protein	5,628	5,011	175	3,420	16.78%
B-12_protein	5,412	4,923	122	3,394	16.65%
SRCM125040_protein	5,285	4,874	99	3,377	16.77%
DSM_319_protein	4,999	4,706	98	3,351	16.44%
IN_103_protein	4,697	4,023	217	3,351	16.44%
Subset_ B					
AB-S79_protein	5,806	5,031	205	4,359	17.08%
NCTC10342_protein	5,727	5,321	58	4,206	16.48%
DSM_32_protein	5,602	5,271	20	4,214	16.51%
PHP1706_protein	5,597	4,938	138	4,296	16.83%
TG1-E1_protein	5,466	4,810	273	4,245	16.63%
WSH-002_protein	5,048	4,675	131	4,208	16.48%

**TABLE 5:** Highlight of *Priestia megaterium* AB-S79 orthologous gene clusters (COG) compared with other closely related *Priestia* species as per OrthoVenn3^*a*^

*Priestia* spp.	Proteins	Clusters	Singletons	Protein relative abundance	Protein absolute abundance
AB-S79_protein	5,806	4,586	246	1,914	12.95%
DSM_25875_protein	5,298	3,643	551	2,034	13.76%
K13_protein	5,265	4,490	148	1,838	12.43%
DSM_27955_protein	5,051	4,161	190	1,819	12.31%
DSM_13796_protein	5,049	4,159	204	1,825	12.35%
SGD-V-76_protein	4,305	3,598	222	1,764	11.93%
DMP08_protein	3,928	3,558	71	1,762	11.92%
CGMCC_protein	3,852	2,672	603	1,825	12.35%

^*a*^ AB-S79_protein (*Priestia megaterium*); DSM_25875_protein (*Priestia abyssalis*); K13_protein (*Priestia aryabhattai*); DSM_27955_protein (*Priestia filamentosa*); DSM_13796_protein (*Priestia endophytica*); SGD-V-76_protein (*Priestia veravalensis*); DMP08_protein (*Priestia flexa*); CGMCC_protein (*Priestia taiwanensis*).

Comparing AB-S79 and closely related *Priestia* spp., 6,397 clusters (5.80% singletons), 198 overlaps, 1,155 single-copy, and 38,554 proteins were highlighted (Fig. 6iii). Of these 1,631 clusters and 14,781 proteins were shared among the *Priestia* spp. (Fig. 6iii). In Table 5, AB-S79 had the highest individual protein (5,806) and cluster count (4,586), while the highest protein (absolute [2,034]; relative [13.76%]) abundance was seen in the DSM_25875 strain. In Fig. 6iv, AB-S79’s closest *Priestia* relative was strain K13_protein (*Priestia aryabhattai*). This result correlates with the Proksee result (Fig. 5A). No gene family variations (expansion and contractions) were generated for these groups by OrthoVenn3. For AB-S79’s relationship with distantly related *Bacillus* spp., 6,310 clusters (7.11% singletons; 2,361), 106 overlaps, 1,325 single-copy, and 33,223 proteins were highlighted (Fig. 6v). Of these 1,881 clusters and 15,225 proteins were shared among *Bacillus* spp. and AB-S79 (Fig. 6v). In Table 6, AB-S79 had the highest individual protein (6,169) and cluster count (4,702), and second highest protein (absolute [2,248]; relative [14.77%]) abundance. AB-S79 also formed a distinct clade as expected but shared most recent ancestry with TG1-6_protein (a *Bacillus cereus* sp.) and ATCC 10792_protein (a *B. thuringiensis* sp.) (Fig. 6vi; Fig. S3).

**TABLE 6:** Overview of *Priestia megaterium* AB-S79 orthologous gene clusters (COG) in relation to other distantly related *Bacillus* species as per OrthoVenn3^*a*^

Closely related *Bacillus* spp.	Proteins	Clusters	Singletons	Protein absolute abundance	Protein relative abundance
AB-S79_protein	5,806	3,537	897	2,458	16.14%
ATCC_10792_protein	6,169	4,702	514	2,248	14.77%
TG1-6_protein	5,263	4,550	127	2,192	14.40%
168168_protein	4,237	3,472	346	2,096	13.77%
ATCC_14580_protein	4,181	3,342	332	2,129	13.98%
GKT04_protein	3,887	3,452	103	2,053	13.48%
FZB42_protein	3,680	3,360	42	2,049	13.46%

^*a*^ AB-S79_protein (*Priestia megaterium*); ATCC_10792_protein (*Bacillus thuringiensis* serovar berliner); TG1-6_protein (*Bacillus cereus*); 168168_protein (*Bacillus subtilis* subsp. subtilis str.); ATCC_14580_protein (*Bacillus licheniformis*); GKT04_protein (*Bacillus amyloliquefaciens*); FZB42_protein (*Bacillus velezensis*).

#### antiSMASH analysis

**Fig 7:**
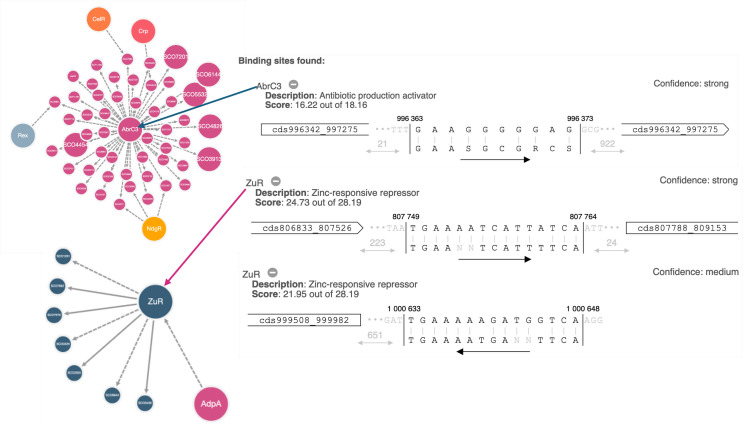
(Top-bottom) Contextual graphical description of TFBS hits with their binding site sequences and the surrounding genes. Top: AbrC3 (confidence: strong); middle: ZuR (confidence: strong); and bottom: Zur (confidence: medium).

**Fig 8:**
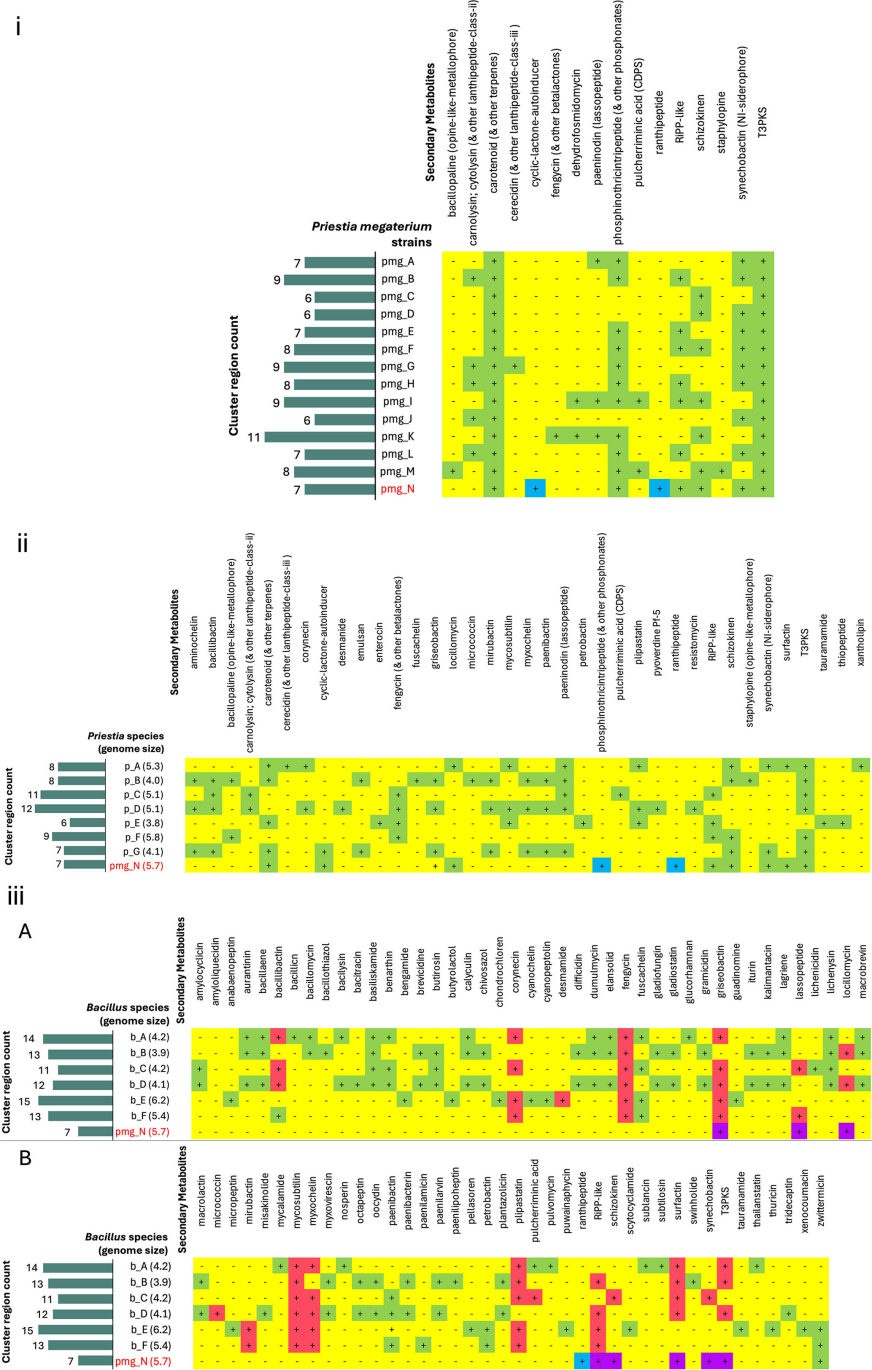
(i) antiSMASH comparison of the biosynthetic gene regions and secondary metabolites among the *Priestia megaterium* “query-genomes14.” The left horizontal bar chart depicts the number of biosynthetic gene cluster regions per strain in the “query-genomes14”; graphical plot highlights the unique secondary metabolites predicted for each *P. megaterium* strain (pmg). Key: pmg_A = WSH-002 (GCF_000225265.1); pmg_B = NCTC10342 (GCA_900445485.1); pmg_C = Q3 (GCF_001050455.1); pmg_D = B-12 (GCA_030270625.1); pmg_E = DSM319 (GCA_029537015.1); pmg_F = NBRC15308 (GCA_001591525.1); pmg_G = DSM_2894 (GCA_029857455.1); pmg_H; DSM32 (GCA_029536995.1); pmg_I = IN103 (GCA_008728535.1); pmg_J = KNU-01 (GCA_024169105.1); pmg_K = PHP1706 (GCA_030548605.1); pmg_L = SRCM125040 (GCA_028548275.1); pmg_M = TG1-E1 (GCA_003074515.1); pmg_N = AB-S79 (GCA_030372795.1). Boxes in green represent secondary metabolite BGCs detected; yellow boxes represent undetected secondary metabolite BGCs; blue boxes represent metabolite BGCs unique to strain AB-S79. (ii) antiSMASH comparison of the biosynthetic gene regions and secondary metabolites among the *Priestia* species. The left horizontal bar chart depicts the number of biosynthetic gene cluster regions and genome size per *Priestia* sp.; graphical plot highlights the unique secondary metabolites predicted per *Priestia* species. Key: p_A = *P. aryabhattai* K13 (GCA_002688605.1); p_B = *P. flexa* DMP08 (GCA_021441905.1); p_C = *P. filamentosa* DSM 27955 (GCA_002237735.1); p_D = *P. endophytica* DSM 13796 (GCA_900115845.1); p_E = *P. taiwanensis* CGMCC 1.12698 (GCA_014638355.1); p_F = *P. abyssalis* DSM 25875 (GCA_002019595.1); p_G = *P. veravalensis* SGDxVx76 (GCA_001457055.1); pmg_*N* = *P. megaterium* AB-S79. (GCA_030372795.1). Boxes in green represent secondary metabolite BGCs detected; yellow boxes represent undetected secondary metabolite BGCs; blue boxes represent metabolite BGCs unique to strain AB-S79. (iii) (**A and B**) antiSMASH comparison of the *P. megaterium* AB-S79 biosynthetic gene regions and secondary metabolites with other distantly related *Bacillus* species. The left horizontal bar chart depicts the number of biosynthetic gene cluster regions and genome size per species; graphical plot highlights the unique secondary metabolites predicted per species. Key: b_A = *B. subtilis* subsp. subtilis str. 168 (GCA_000009045.1); b_B = *B. velezensis* FZB42 (GCA_000015785.2); b_C = *B. licheniformis* ATCC 14580 (GCA_034478925.1); b_D = *B. amyloliquefaciens* GKT04 (GCA_019396925.1); b_E = *B. thuringiensis* serovar berliner ATCC 10792 (GCA_000161615.1); b_F = *B. cereus* TG1-6 (GCA_003013315.1); pmg_*N* = *P. megaterium* AB-S79 (GCA_030372795.1). Boxes in green represent secondary metabolite BGCs detected; red boxes represent secondary metabolite BGCs in *Bacillus* members shared with *Priestia* members; yellow boxes represent undetected secondary metabolite BGCs; purple boxes represent those present in *P. megaterium* members; blue boxes represent secondary metabolite BGCs unique to *P. megaterium* AB-S79.

antiSMASH predicted seven biosynthetic cluster regions in the *P. megaterium* AB-S79 genome (Table 7). Also, two regulators, zinc-responsive repressor (Zur; region 33.1) and antibiotic production activator (AbrC3; regions 33.2 and 42.1), showed strong prediction threshold in the transcription factor binding site (TFBS) finder profile of antiSMASH (Fig. 7 [top-bottom]). Zur also showed medium prediction thresholds in regions 23.3, 33.1, and 33.2 (Fig. 7 [bottom]). The other regulators like the regulator of arginine biosynthesis genes (ArgR; regions 23.1 and 33.1), NAD synthesis repressor (NrtR; region 44.1), development and antibiotic global regulator (BldD; regions 33.2 and 44.1), and cellobiose uptake repressor (CelR; regions 33.1 and 44.1) had weak predictions (Fig. S4i [top-bottom]). While AB-S79’s genome boasts of gene clusters for biosynthesizing terpenes and synechobactin, the discovery of the schizokinen and ranthipeptide genes in regions 33.1 and 44.1 further piqued our attention (Fig. S4ii [top-bottom]). Compounds like carotenoids, phosphonates, synechobactins, and schizokinens were common among the *P. megaterium* strains. BGCs belonging to a cyclic-lactone-autoinducer and ranthipeptide were, however, specific to AB-S79 (Fig. 8i). Although carotenoids, synechobactins, and schizokinens were shared also among some of the *Priestia* spp. including AB-S79, phosphonate and ranthipeptide remained exclusive to AB-S79 (Fig. 8ii). As ranthipeptide (BGC) was exclusive to AB-S79 and undetected in the *Bacillus* genomes, compounds like mycosubtilin, myxochelin, and plipastatin were sparsely shared among some of the *Bacillus* and *Priestia* sp. genomes while completely undetected in AB-S79 (Fig. 8iii).

**TABLE 7:** antiSMASH predicted biosynthetic regions and secondary metabolites in the *Priestia megaterium* AB-S79 genome^*a*^

Region	Gene Cluster type	From	To	Most similar known cluster	Similarity (percentage (%))
Region 23.1	Terpene	20,397	41,245	Carotenoid	Terpene (50%)
Region 23.2	Phosphonate	163,267	180,691	Phosphonate	Unspecified
Region 23.3	T3PKS	824,645	865,730	Uncharacterized	Unspecified
Region 33.1	NI-siderophore	796,653	831,228	Synechobactins	Other (23%)
Region 33.2	Terpene	980,260	1,001,078	Squalene/phytoene	Other (14%)
Region 42.1	Terpene	494,956	516,824	Uncharacterized	Unspecified
Region 44.1	Ranthipeptide	1	18,346	Uncharacterized	Unspecified

^*a*^ T3PKS, type 3 PKS (polyketide synthase); NI-siderophore, NRPS-independent, IucA/IucC-like siderophores.

#### Geneious Prime exploration

**Fig 9:**
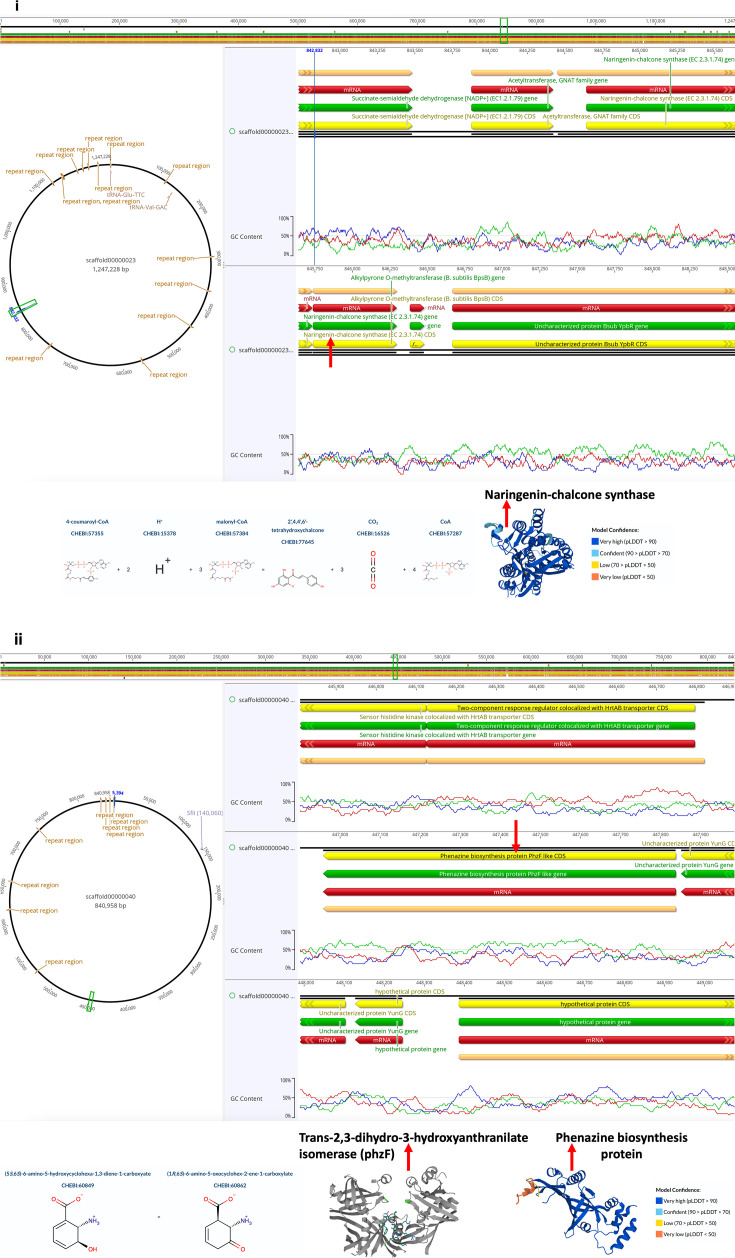
(i) Annotation of naringenin-chalcone synthase biosynthetic cluster (v.2024.0.4), catalytic activity, and structure. Protein structure was inferred from homology on Uniprot (https://www.uniprot.org/). (ii) Annotation of phenazine biosynthesis protein PhzF like (v.2024.0.4), catalytic activity, and structure. Protein structure was inferred from homology on Uniprot (https://www.uniprot.org/).

Geneious Prime probing revealed four unique biomolecules: (i) naringenin-chalcone synthase (loci: 844,645–845,730); (ii) dienelactone hydrolase family protein (loci: 497,927–498,529); (iii) phenazine biosynthesis protein PhzF like (loci: 446,959–447,837 and 509,962–510,852); and (iv) kynurenine pathway enzymes (kynureninase and kynurenine formamidase) (loci: 691,495–692,781; 695,268–695,894; and 1,048,914–1,049,582) (Fig. 9i and ii; Fig. S5i and ii).

#### BV-BRC analysis

BV-BRC analysis revealed 57 key pathway classes in *P. megaterium* AB-S79 (Table S2). Of these 57 pathway classes, 5 correlated with biosynthesis of polyketides and nonribosomal peptides, 24 with biosynthesis of secondary metabolites, 7 with energy metabolism, and 21 with xenobiotics biodegradation and metabolism. Notable pathways include those for the biosynthesis of ansamycins and puromycin, flavone and flavonol biosynthesis (naringenin), terpenoid backbone biosynthesis, brassinosteroid biosynthesis, and several biodegradative pathways (Table S2).

**Fig 10:**
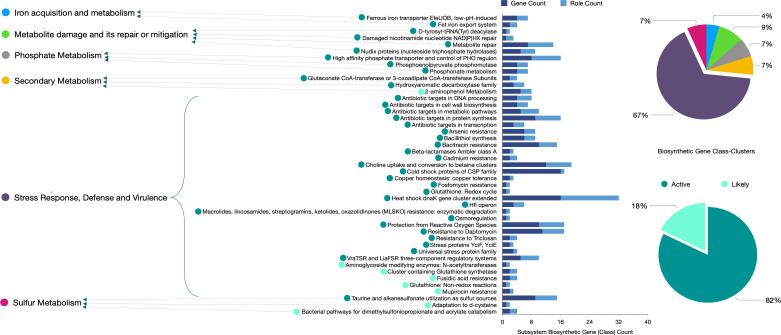
BV-BRC’s detailed biosynthetic gene (BG) distribution across the 45 selected *P. megaterium* AB-S79 BG class clusters. Bar charts show subsystem biosynthetic gene (class) and role count; pie charts detail the percentage of active and inactive biosynthetic gene class clusters.

BV-BRC analysis unveiled 45 key BG class clusters linked with (i) iron acquisition and metabolism, (ii) metabolite damage and its repair or mitigation, (iii) phosphate metabolism, (iv) secondary metabolism, and (v) stress response, defense, virulence, and sulfur metabolism. In total, 203 individual BGs were distributed across the 45 selected BG class clusters; within these BGs class clusters, 38 (82%) were termed active while 8 (18%) were termed likely active (Fig. 10). Among the 549 pathway classes, secondary metabolite biosynthesis and the immune system pathway had the highest and lowest proportions, respectively (Fig. S6i). The cofactors, vitamins, and prosthetic groups had the most BG class clusters of the total 2,149 BG class clusters as per BVRC annotations While the cell envelope, capsule, and slime layer groups had the least BG class cluster (Fig. S6ii).

## DISCUSSION

Genome prospecting and bioinformatics (32, 45, 46), including web-based open-source technologies, have reshaped the quest for microbial value-added products (47, 48). Web-based bioinformatic database systems have also become key tools for investigating genomic evolution and expression across species (31, 49). Among these species, *Priestia megaterium* has the metabolic capacity to produce useful biomolecules like enzymes and recombinant proteins (4). This is the first genome-scale comparative study of *P. megaterium* spp. since its reclassification, and it should guide future research into the species’ evolutionary trends, genetic relatedness, and geographic distribution. In Fig. 1, we show the graphical approach for our *in silico* web-based investigation of the *P. megaterium* AB-S79 genome, which could be applied to other prokaryotic web-based genome mining studies. Genome statistics of “query-genomes14” in Table 1 and *P. megaterium* AB-S79’s subsystem exemplifies AB-S79’s robust genomic profile with a cross-section of proteins with functional assignments, EC number assignments, GO assignments, pathway assignments, and PATRIC genus-specific family and cross-genus family (PGfam) assignments (Tables 2 and 3). AB-S79’s protein profiles and specialist genes classify it as a significant *P. megaterium* lineage.

Kbase Compute Pangenome software enabled the grouping of all protein-coding genes in the imputed “query-genomes14” into core protein families and singleton (non-core and non-singleton fraction) based on their sequence homology (50, 51). The homolog families showed consistency across the “query-genomes14” (Fig. 3A), confirming that *P. megaterium* is a highly conserved group of species with gene overlaps and comparable traits (Fig. 3B). Contrastingly, for the homolog genes, genomes of *P. megaterium* PHP1706 (GCA_030548605.1), *P. megaterium* KNU-01 (GCA_024169105.1), and *P. megaterium* SRCM125040 (GCA_028548275.1) were the outliers. Core genes exist across all imputed genomes in a pangenome (51); hence, every strain in the “query-genomes14” would have inherited or harbor comparable ancestral genes. Because of their roles in ecological survival and lifestyle, core genes are expected to be retained across species’ lineages (49, 52). The AB-S79 singletons (Fig. 3A) share no sequence homology with genes in any other genome since singletons occur in one and only one genome (36, 52). They may be acquired horizontally from distal lineages (52), and/or be related to recent environmental adaptations (53, 54). Kbase’s analysis of *P. megaterium* populations here reveals that the species share evolutionary commonality, as demonstrated in prior studies (55).

In the phylogenetic tree (Fig. S1A and B), AB-S79 clustered closely with *P. megaterium* DSM 319, a soil isolate from the USA. Comparing host groups, genomes isolated from plants had the largest percentage (112 [32%]) (Fig. 4C), correlating with *P. megaterium* sp. plant growth promotion roles reported previously (4, 11). The global distribution of the genus *Priestia megaterium* reveals that Africa (Fig. 4D) has a low genomic output—our native isolate has propensity for future biotechnological evaluation within the taxa. According to the GBDP phylogeny tree and GTDB phylogeographic tree (Fig. 5A and B), AB-S79 clusters closely with the type strain *P. megaterium* NBRC 15308 = ATCC 14581 obtained from Japan. As with previous report (55), this study reveals a close phylogenetic relationship between *P. megaterium* and *P. aryabhattai* (Fig. 5A). Overall, the results of the phylogenomic analyses conducted in this study using multiple databases were comparable.

Comparing orthologs is important in comparative genomics for investigating evolutionary relationships traversing genome structure, gene function, and taxonomic classification across different organisms (56–58). Orthologous sequences in different species may serve either equivalent biological roles or alternative functions in single species (59). By OrthoVenn3’s inference, AB-S79 genome had the most protein, singleton, and protein absolute abundance among the six *P*. *megaterium* strains (Fig. 6i; Table 4), suggesting that AB-S79 has the most evolutionary shift. Invariably, AB-S79 would harbor more unique functional genes, and its BGCs would retain and/or exhibit functions comparable to those of its parent. Contrastingly, in Fig. 6ii and Table 4 (Subset_B), AB-S79’s cluster and singleton counts dipped as against other *P. megaterium* strains. Regardless, AB-S79 contained 9–10 unique homologous clusters dedicated to biological processes, molecular functions, and cellular components (Fig. 6i and ii). Furthermore, as shown in the expansions and contractions (Fig. S1A, B and Fig. S2i through iv), each *P. megaterium* distinguished itself despite their evolutionary proximity to one another, thus cementing their statuses within the taxa. Comparing the *P. megaterium* strains investigated in this study, AB-S79 had the most evolutionary shifts.

In previous reports by Chandra et al. (60) and Khalifa and Alsowayeh (61), *P. megaterium* and *P. aryabhattai* contained more coding genes than other *Priestia* members. Here, AB-S79 also had the highest protein count of any *Priestia* members (Table 5), closely followed by *P. aryabhattai* K13, its closest *Priestia* relation (Fig. 6iv). Thus, predictably, AB-S79 should have greater genomic complexity, functional diversity, evolutionary divergence, environmental adaptation, or advanced gene regulatory mechanisms than other *Priestia* members analyzed in this study. These aforementioned attributes were not areas we fully explored in this study. Also, since no gene family variations (expansion and contractions) were generated for the *Priestia* group by OrthoVenn3, we could not explore the level of evolutionary changes that occurred among *Priestia* strains analyzed in this study.

On the other hand, we anticipated some genetic relatedness and evolutionary relationship between AB-S79 and the *Bacillus* spp. investigated in this study, since *Priestia* sp. was previously categorized under the genus *Bacillus* (until two conserved signature indels (CSIs) in two peptide sequences from the desert hedgehog (DHH) superfamily of peptides were identified) (9). Based on Fig. 6vi and Fig. S3, AB-S79 clustered separately from the *Bacillus* strains forming a distinct clade but shared most recent ancestry with TG1-6_protein (a *Bacillus cereus* sp.) and ATCC 10792_protein (a *B. thuringiensis* sp.). With AB-S79 having the greatest expansion and contraction ratio (+123/–177) relative to its distant *Bacillus* relations, the strain would have undergone the most evolutionary shift (Fig. 6vi). In species, genome expansions and contractions can be aided by increased genetic drift, selection, or entirely neutral processes (62). OrthoVenn3’s contraction and expansion analysis of gene families reveals significant changes in the evolution of the genomes under study in relation to our isolate AB-S79. The expansion and contraction of gene families in the examined genomes are indicative of the evolutionary pressures they have undergone including, horizontal gene transfer, gene duplication, and/or loss (63). These variations would be expected to influence their metabolic flexibilities, survivability, and interactions in their environments (64)].

In their study of *Bjerkandera adusta*, Moody et al. (65) found that the relative abundance of each specific protein was proportional to the protein amounts identified as participating in specialized metabolism and xenobiotic prevention. Protein abundance is the amount of copies of a protein molecule in a cell (66). A species’ protein relative abundance also reveals the protein’s rarity in contrast to other species in a given setting. Protein levels have a direct impact on cellular processes and molecular traits, leading to heterogeneity across species (67, 68). Considering that AB-S79 had the most protein relative and absolute abundance, and the highest proportion of singletons compared to its distant *Bacillus* relatives (Table 6), we conclude that the protein fractions in AB-S79 involved in specialized metabolism would supersede those in its distant *Bacillus* relatives. This study did not explore the individual biological process, molecular function, cellular component, and gene ontology enrichment cluster categories of the genomes analyzed using OrthoVenn3, as it would deviate from the main objective of the study.

The presence of global and pathway-specific regulators in secondary metabolite-producing microbes is associated with active BGCs (29). antiSMASH’s detection of the two regulators, AbrC3 and Zur, with strong prediction threshold in the TFBS finder profile affirms the active state of AB-S79’s BGCs and its metalloregulatory potential (Fig. 7 [top-bottom]; Fig. S4i [top-bottom]). The two-component system molecule AbrC3 is similar to the NarL family of genes in *Streptomyces coelicolor* (69, 70), functioning as a positive response regulator of antibiotic production (70, 71). Conversely, the multifunctional response regulator Zur belongs to the Fur gene family and is notable for modulating zinc assimilation in diverse bacteria species (72, 73).

antiSMASH detected BGCs for terpenes (region 23.1, 33.2, and 42.1), phosphonate (region 23.2), siderophores (synechobactins and schizokinens [region 33.1]), and ranthipeptide (region 44.1) in AB-S79 (Table 7; Fig. S4ii [top-bottom]). Excluding ranthipeptide, BGCs for the other five metabolites appear to be conserved in all the *P. megaterium* genomes analyzed in this study (Fig. 8i). Terpene synthases (e.g., squalene and phytoene synthase) in AB-S79 and other microorganisms are involved in the mevalonate pathway biosynthesis of terpenoids (74). Terpenoids also classified in the BV-BRC’s analysis (Table S2) are important natural products with biomedical and pharmaceutical applications (75, 76). The siderophores schizokinen and synechobactin in AB-S79 are produced by marine cyanobacteria like *Synechococcus* sp. PCC 7002, in response to low iron concentrations (77, 78). Schizokinen, first described in *Bacillus megaterium* ATCC 19213 (79), comprised of citric acid symmetrically substituted by amide linkages to a pair of 1-amino-3-(N-hydroxy-N-acetyl)-aminopropane residues (80, 81). Previous reports confirm metal chelation by *B. megaterium* ATCC 19213’s schizokinen through the siderophore transport receptor (82). Siderophores are popular for the competitive advantage they confer on producing microbes (83). Notably, schizokinens and synechobactin were sparsely dispersed in the other *Priestia* and *Bacillus* genomes, indicating an evolutionary correlation between them and AB-S79 (Fig. 8i through iii).

Phosphonates are organophosphorus biomolecules found abundantly in marine habitats (84, 85). Their degradation substantially contributes to ecosystem function, and they are critical for marine phosphorus (P) and global biogeochemical P cycling (86–88). Phosphonate BGs are dispersed in many bacteria and archaea; however, they are only biosynthesized sporadically in producing strains (86, 88). Many phosphonate-producing genomes lack genes required for its dissimilation and synthesis (86, 89); AB-S79 phosphonate BGCs are most likely for phosphonate dissimilation. According to Romano (90) and Acker et al. (86), horizontal acquisition of phosphonate BGs appears predominant. Thus, ABS-79’s phosphonate BGCs, as with other *P. megaterium* strains here (Fig. 8i), are likely horizontally acquired. Although Wilson et al. (91) reported phosphonate BGC discovery in a *Bacillus velezensis* genome, here, phosphonate BGCs seem exclusive to the *P. megaterium* sp., juxtaposed with other *Priestia* and *Bacillus* members (Fig. 8ii and iii) in this study. While antiSMASH detected bacillibactin, mycosubtilin, fengycin, corynecin, mirubactin, and plipastatin conjointly in the *Bacillus* and *Priestia* genomes (Fig. 8iii [A and B]), the secondary metabolites concurrently found in the *Bacillus* and *P. megaterium* genomes were griseobactin, lassopeptide, synechobactin, and schizokinens. Furthermore, antiSMASH predicted no secondary metabolite BG regions in 44 additional nodes of the AB-S79 genome. These undefined 44 nodes may represent clusters possessing novel undefined functions.

The BGCs for naringenin-chalcone synthase, dienelactone hydrolase family protein, phenazine biosynthesis protein PhzF like, and kynurenine pathway enzymes (kynureninase and kynurenine formamidase) discovered in the ABS-79 genome by Geneious Prime probing (Fig. 9i and ii; Fig. S5i and ii) suggest ABS-79’s relevance in the metabolism of these biomolecules’ parent compounds. The metabolism of naringenin (a versatile bioactive flavanone polyphenol) (92) by other *P. megaterium* strains has been reported (93, 94). Naringenin chalcone synthase catalyzes the condensation of tyrosine-derived *p*-coumaric acid and three malonyl-CoA units to synthesize the naringenin chalcone (95) (Fig. 9i). The dienelactone hydrolase family protein (Fig. S5i) includes dienelactone hydrolase (carboxymethylenebutenolidase), an alpha (α)/beta (β) protein that catalyzes the hydrolysis of dienelactone to maleylacetate, a tricarboxylic acid cycle substrate during the microbial degradation of chloroaromatics via chlorocatechols pathway (96–98). Xenobiotic degraders from *Burkholderia* sp., *Rhodococcus* sp., *Pseudomonas* sp., and *Sphingomonas* sp. produce dienelactone hydrolase family protein like AB-S79 (99–101).

Studies on the distribution and evolution of phenazine genes infer that many phenazine-producing species are soil inhabitants (102), and interdomain gene transfer (e.g., bacterial-fungi transfer) does occur (103). The detection of phenazine biosynthesis protein PhzF like (Fig. 9ii) in our soil isolate ABS-79 genome correlates with the above studies. The enzyme’s role in ABS-79 is likely for trans-2,3-dihydro-3-hydroxyanthranilic acid metabolism (104, 105) and phenazine biosynthesis (106, 107). Phenazines and phenazine-containing compounds produced by various bacteria have multiple functions and biotechnological uses (107, 108). Discovering kynureninase and kynurenine formamidase (Fig. S5ii) in the AB-S79 genome suggests ABS-79 could be relevant in kynurenine metabolism. The kynurenine pathway, a primary route for tryptophan catabolism in many organisms (109), is key in cellular energy generation in nicotinamide adenine dinucleotide (NAD^+^) form (110), thus contributing to immune system modulation (109, 111). Kynureninase is a pyridoxal phosphate (PLP)-dependent enzyme catalyzing the cleavage of kynurenine into anthranilic acid (109, 112). Anthranilic acid and its analogs have industrial and medicinal use (113). Kynurenine formamidase likewise catalyzes the second step of the kynurenine-NAD^+^ biosynthetic pathway by hydrolyzing *N*-formyl kynurenine to produce kynurenine and formate (114).

Considering the spectrum of metabolic resources unveiled by BV-BRC’s analysis dedicated to stress response, defense response, virulence, metabolite damage, and repair by AB-S79, it is evident AB-S79 prioritizes environmental survivability. In AB-S79’s subsystem, for example, heat and cold shock proteins exhibited a high gene count. Based on the role count, the heat shock proteins appear to play a critical role in AB-S79’s subsystem. Dispersed across AB-S79’s genome are pathway class clusters conferring resistance to diverse recalcitrant compounds which correlates with its *in vitro* multi-metal-resistant traits. By implication, AB-S79’s BGCs associated with xenobiotic biodegradation are active (Table S2; Fig. 10). Pathway classes notable for producing biomolecules like puromycin, flavone (naringenin), novobiocin, anthocyanin, thermolysin, ankyrin, and butirosin were also highlighted in the BV-BRC’s analysis. Some of these relevant compounds have been investigated previously (115–119). Alongside antiSMASH, BV-BRC analysis revealed that the AB-S79 genome included putative proteins without clearly defined biological roles warranting further investigation.

### Conclusion

In an era of declining discovery rates of novel biomolecules, genome mining technology offers enormous promise to rejuvenate the natural product discovery pipeline and help overcome the current obstacles in the discovery of new bioactive molecules (e.g., antibiotics). Comparative genomics studies offer deeper insights into the distribution of genes, proteins, and pathways among various microbial species, indicating their significance in genome evolution and adaptation, and paving the way for their biotechnological exploitation. This study underscores the enduring value of microbial natural product discovery strategies (e.g., genome mining). Given that the *P. megaterium* sp. encodes most genes for natural competence formation and shares traits with other microbial cell factory models (4), this study highlights not just the potential of a single genome of the species as a biomolecule storehouse, but also that of other members of the taxon. Notwithstanding the study constraints, the conclusions drawn from this study showcase *P. megaterium* AB-S79’s distinct genetic features and biotechnological potential. This study helped to (i) assess the extent of gene conservation among the imputed *P. megaterium* genomes, (ii) understand *P. megaterium* AB-S79’s evolutionary history, and (iii) identify unique biomolecules in the isolate’s genome. To fully appreciate and exploit relevant microorganisms such as *P. megaterium* AB-S79, interdisciplinary multi-omics research will be necessary.

## Data Availability

The *P*. *megaterium* AB-S79 draft whole-genome shotgun project was deposited in DDBJ/ENA/GenBank and is available under accession number JAUCND010000023.1.

## SUPPLEMENTAL MATERIAL

Supplemental figures spectrum.01466-24-s0001.pdf Fig. S1 to S6.

Supplemental tables spectrum.01466-24-s0002.pdf Tables S1 and S2.

ASM does not own the copyrights to Supplemental Material that may be linked to, or accessed through, an article. The authors have granted ASM a non-exclusive, world-wide license to publish the Supplemental Material files. Please contact the corresponding author directly for reuse.
