# Supplementary material for: Unveiling novel features and phylogenomic assessment of indigenous Priestia megaterium AB-S79 using comparative genomics
Source: Microbiol Spectr. 2025 Feb 19;13(4):e01466-24. doi: 10.1128/spectrum.01466-24 (PMC11960082; doi:10.1128/spectrum.01466-24)
Supplement: Supplemental figures — Fig. S1 to S6. [file spectrum.01466-24-s0001.pdf]

Supplementary Figures

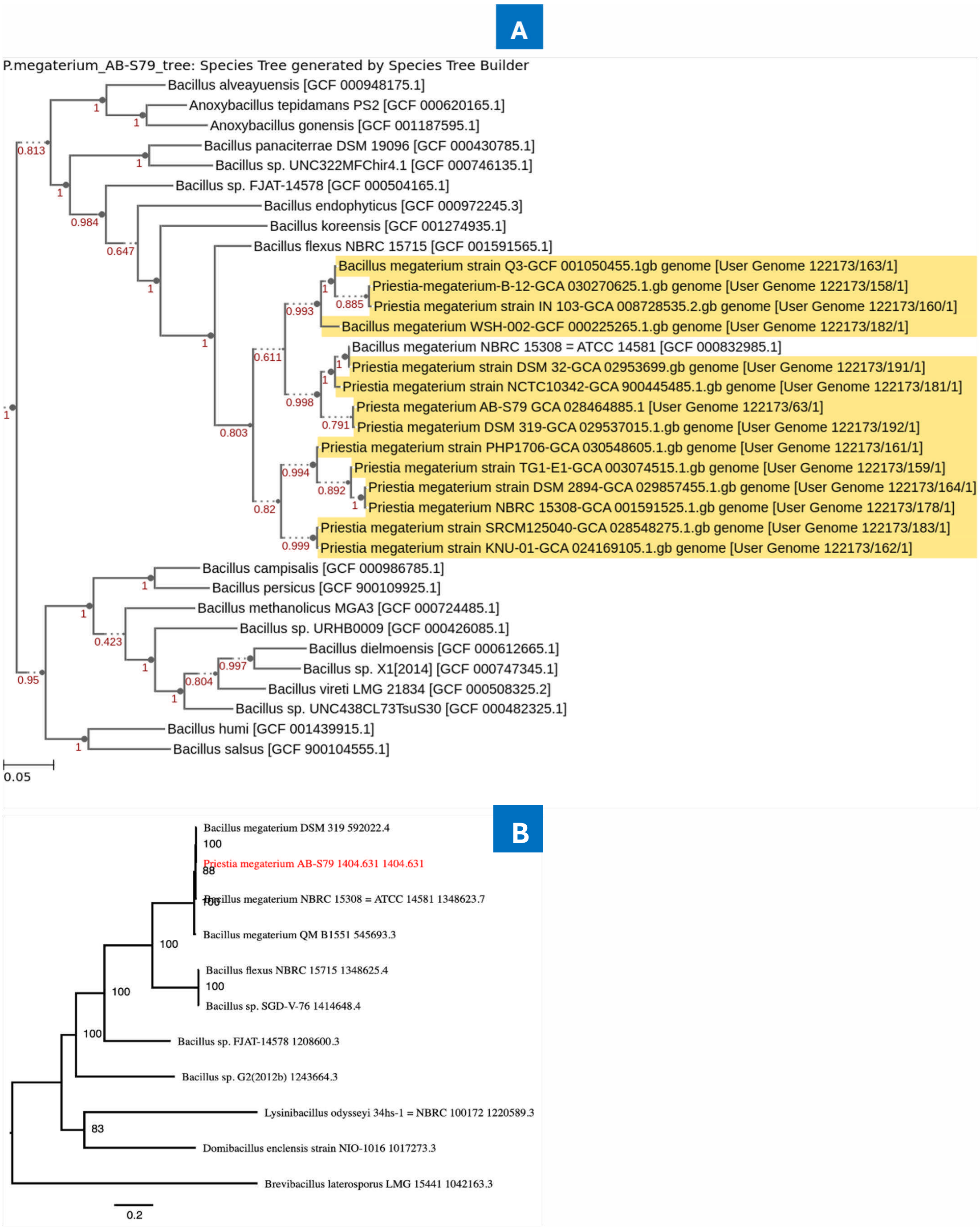

Figure S1 (A & B), Phylogenetic trees of whole-genome sequencing data of previously published *Priestia megaterium* strains and *P. megaterium* AB-S79 genome. **A.** Kbase *P. megaterium* AB-S79 phylogenetic tree; **B.** BV-BRC *P. megaterium* AB-S79 phylogenetic tree).

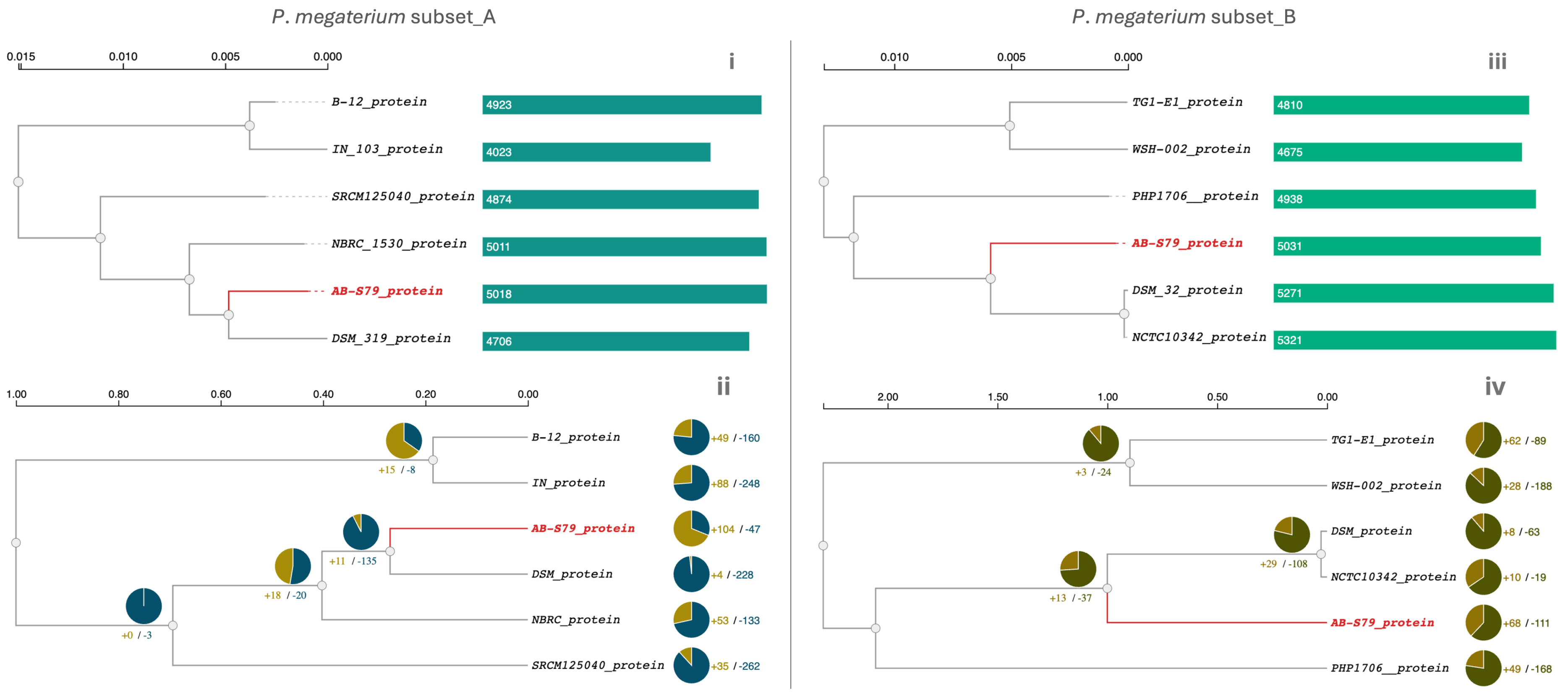

Figure S2 (i–iv): OrthoVenn3 trees describing the evolutionary timelines and relationship between the 'query-genomes14' subsets (Subset_A and Subset_B) based on the identification of highly conserved single-copy genes. **Left panel i. & ii.** Phylogenetic and gene family (expansion and contraction) trees of AB-S79 with Subset_A of 'query-genomes14' (expansion (lemon) and contraction (teal)); **Right panel iii & iv**. Phylogenetic and gene family (expansion and contraction) trees of AB-S79 with Subset_B of 'query-genomes14' (expansion (lemon) and contraction (green)). Genome size of the each strain is detailed in the bar charts.

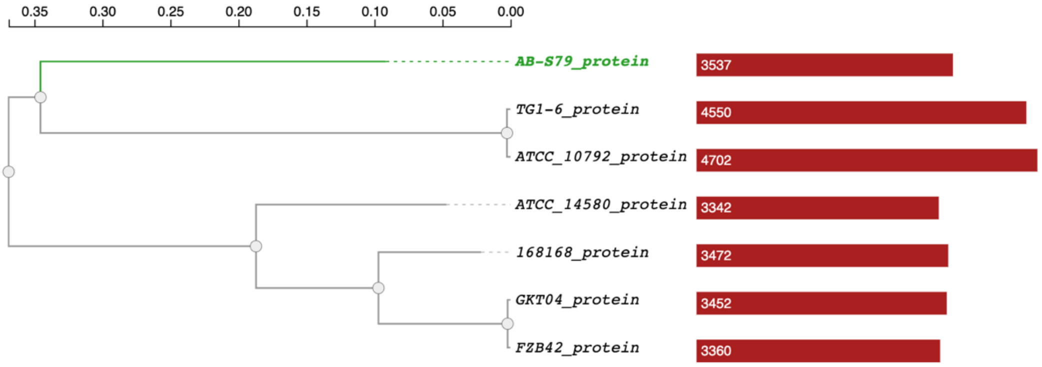

Figure S3: OrthoVenn3 phylogenetic tree describing the evolutionary timelines and relationships between *P. megaterium* AB-S79 and its distantly related type strains of the *Bacillus* genus based on the identification of highly conserved single-copy genes. Genome size of the each strain is detailed in the bar charts.

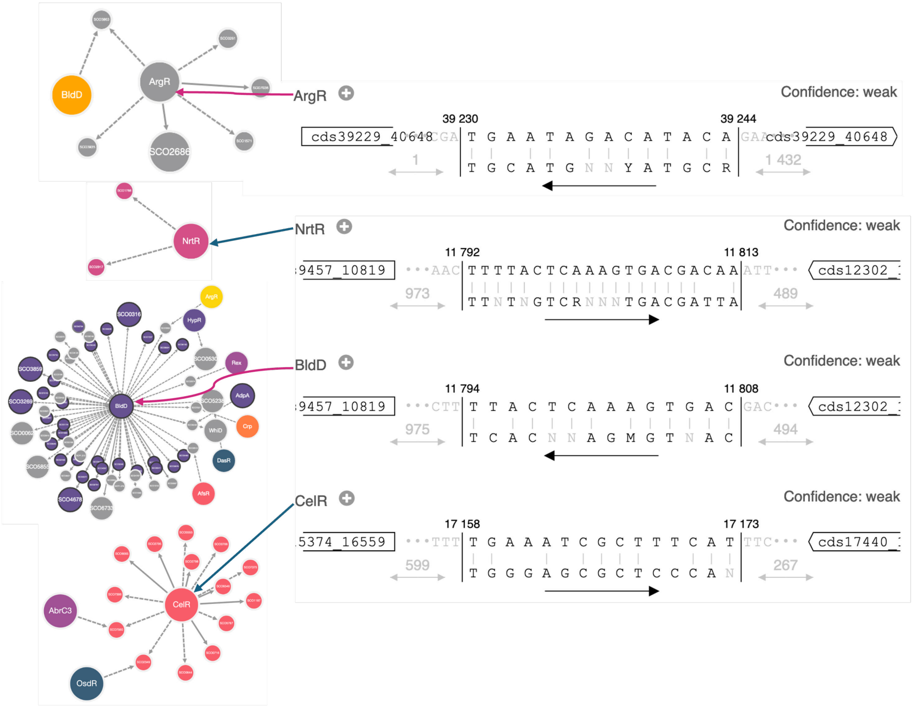

Figure S4i (top-bottom): Contextual graphical description of *Priestia megaterium* AB-S79 genome strong transcription factor binding sites (TFBS) hits with their binding site sequences and surrounding genes.

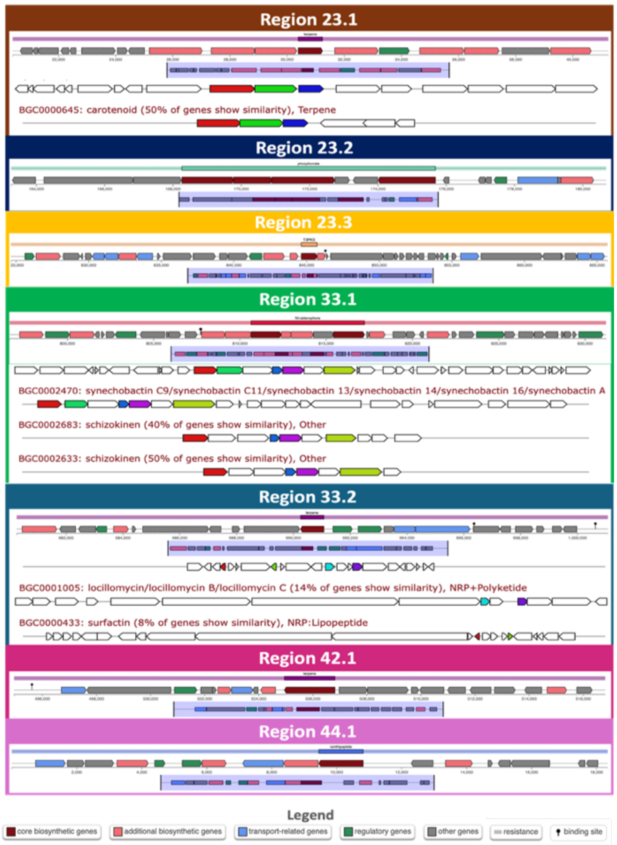

Figure S4ii (top-bottom): Biosynthetic gene cluster similarity profile for the predicted secondary metabolites in the *Priestia megaterium* AB-S79 genome by antiSMASH.

ii

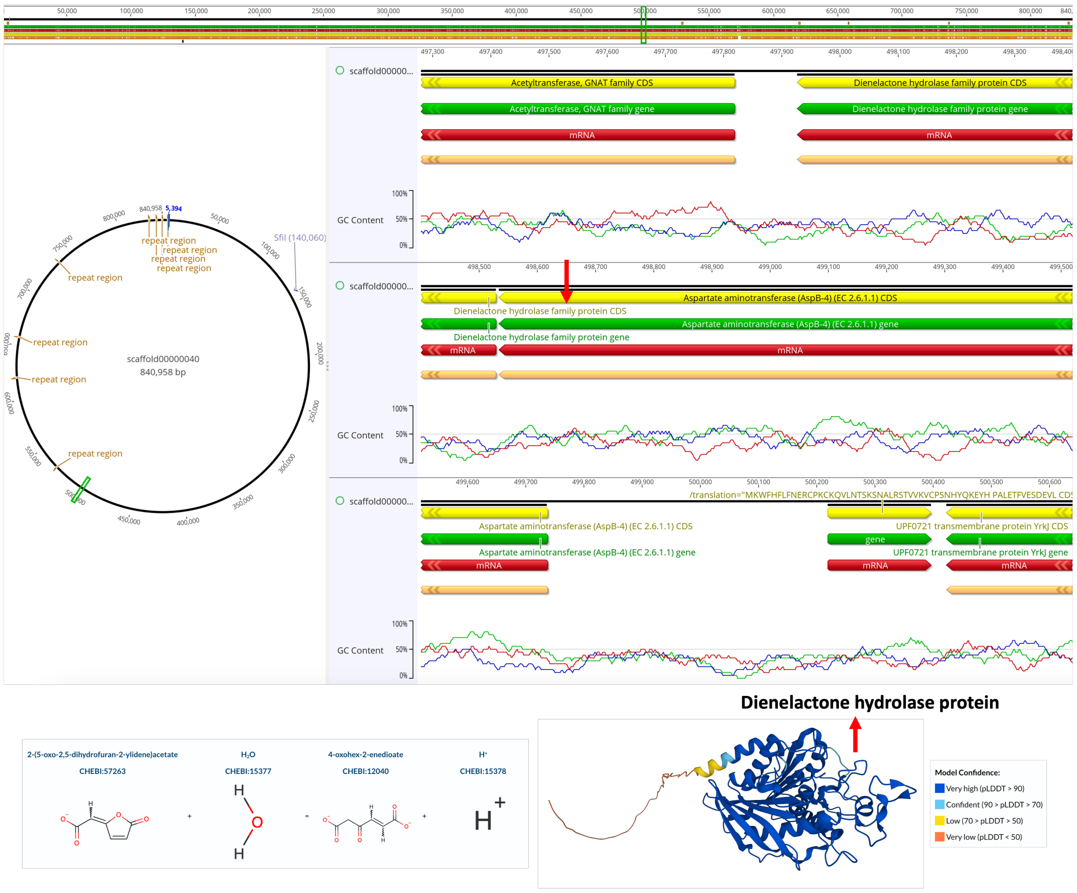

Figure S5i: Annotation of *Priestia megaterium* AB-S79 dienelactone hydrolase family protein (v2024.0.4), catalytic activity, and structure. Protein structure was inferred from homology on Uniprot (https://www.uniprot.org/)).

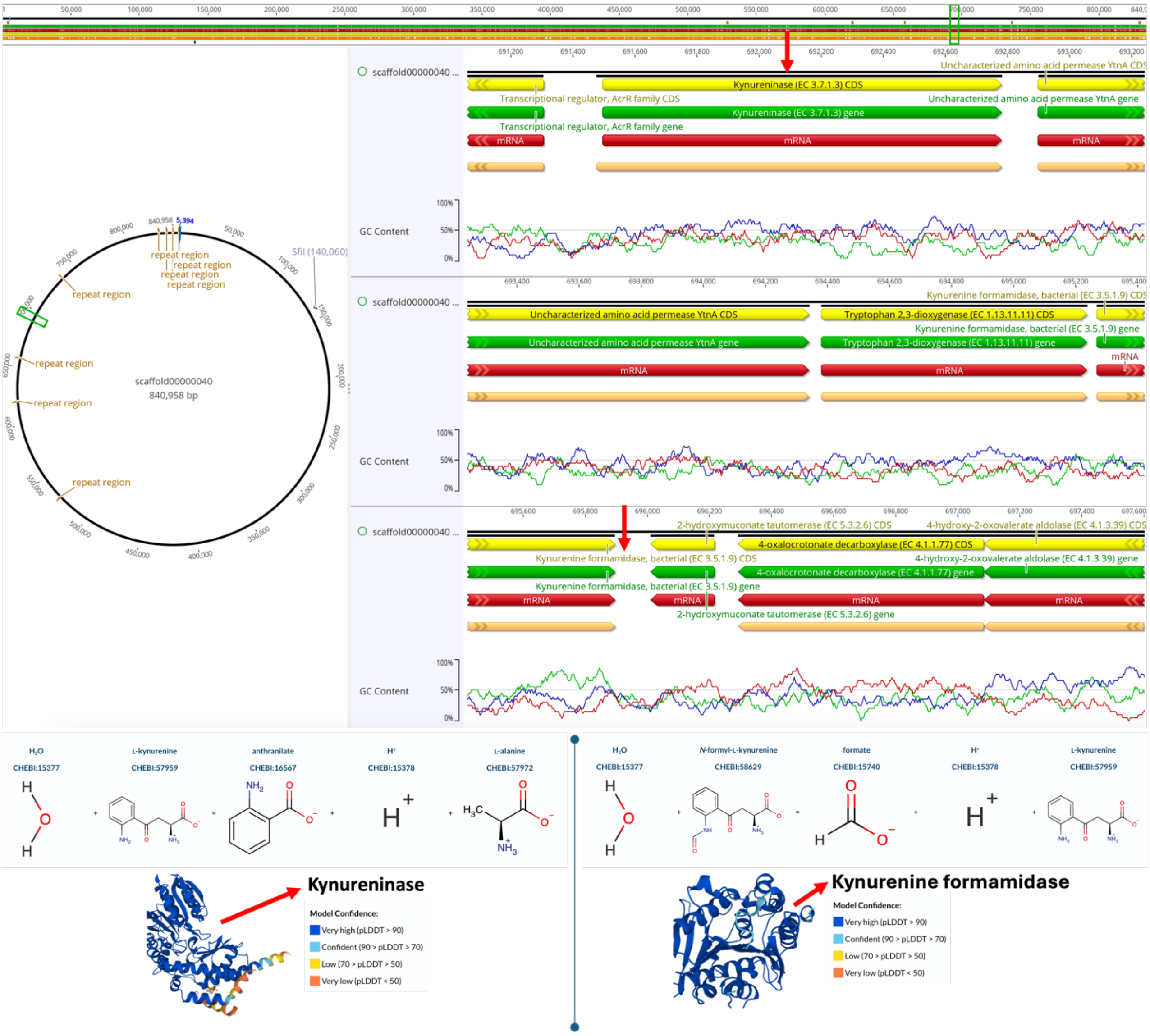

Figures S5ii: Annotation of *Priestia megaterium* AB-S79 genome kynurenine pathway enzymes (kynureninase and kynurenine formamidase) (v2024.0.4), catalytic activity, and structure. Protein structure was inferred from homology on Uniprot (https://www.uniprot.org/)).

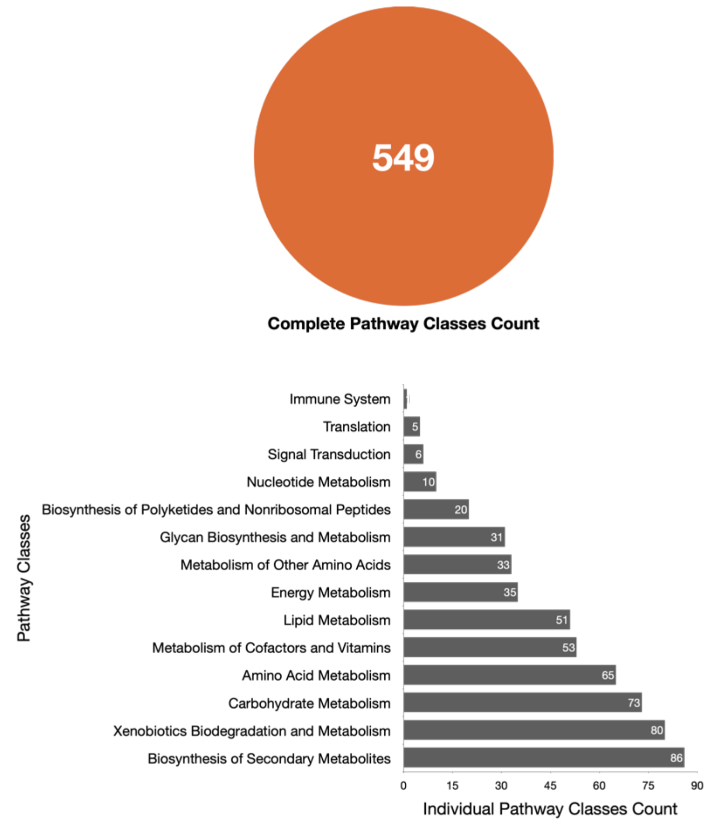

Figure S6i: Highlight of the complete *Priestia megaterium* AB-S79 genome pathways and pathway class counts.

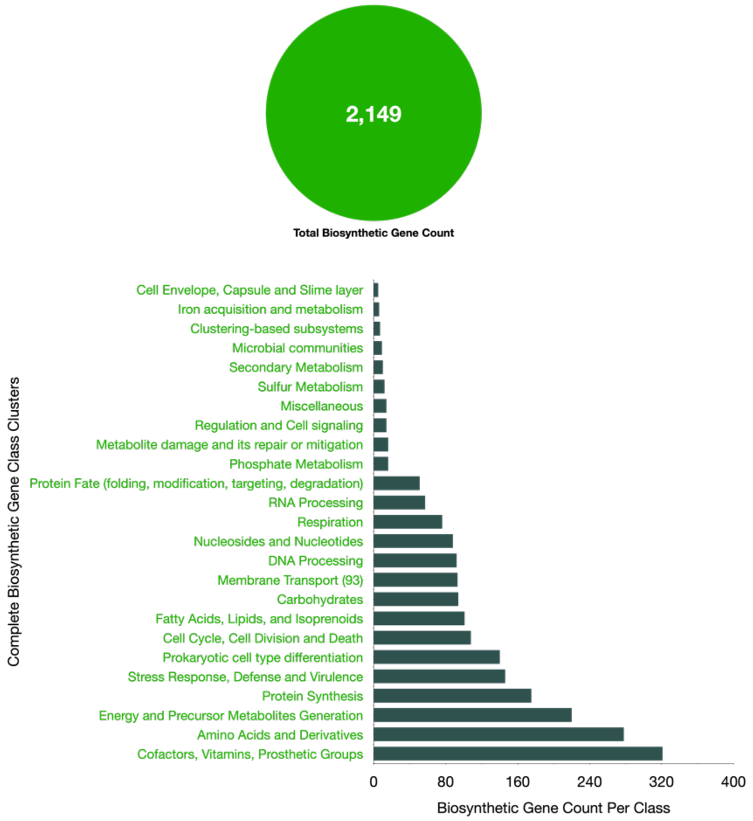

Figure S6ii: Highlights of the complete *Priestia megaterium* AB-S79 genome biosynthetic genes (BGs) within the BG class-clusters as per BVRC annotations.
