# Supplementary material for: Unveiling novel features and phylogenomic assessment of indigenous Priestia megaterium AB-S79 using comparative genomics
Source: Microbiol Spectr. 2025 Feb 19;13(4):e01466-24. doi: 10.1128/spectrum.01466-24 (PMC11960082; doi:10.1128/spectrum.01466-24)
Supplement: Supplemental tables — Tables S1 and S2. [file spectrum.01466-24-s0002.pdf]

Supplementary Tables

Table S1. Antimicrobial Resistance Genes Profile of *Priestia megaterium* AB-S79 Genome

**AMR Mechanism**	**Genes**
Antibiotic inactivation enzyme	FosB, Vgb(A)
Antibiotic target in susceptible species	Alr, Ddl, dxr, EF-G, EF-Tu, folA, Dfr, folP, gyrA, gyrB, inhA, fabI, Iso-tRNA, kasA, MurA, rho, rpoB, rpoC, S10p, S12p
Antibiotic target protection protein	BcrC
Antibiotic target replacement protein	fabL
Efflux pump conferring antibiotic resistance	BceA, BceB
Gene conferring resistance via absence	gidB
Protein-altering cell wall charge conferring antibiotic resistance	GdpD, PgsA
Protein modulating permeability to antibiotic	OprD family
Regulator modulating expression of antibiotic resistance genes	BceR, BceS, LiaF, LiaR, LiaS

Table S2: BV-BRC's report of the beneficial pathway classes within the *P. megaterium* AB-S79's genome

Pathway Name (ID)	Pathway Class	Annotation
1. Biosynthesis of 12-, 14- and 16-membered macrolides (522)<br>2. Biosynthesis of ansamycins (1051)<br>3. Biosynthesis of siderophore group nonribosomal peptides (1053)<br>4. Biosynthesis of type II polyketide backbone (1056)<br>5. Biosynthesis of type II polyketide products (1057)<br>6. Puromycin biosynthesis (231)<br>7. Caffeine metabolism (232)<br>8. Tetracycline biosynthesis (253)<br>9. Penicillin and cephalosporin biosynthesis (311)<br>10. beta-Lactam resistance (312)<br>11. Novobiocin biosynthesis (401)<br>12. Streptomycin biosynthesis (521)<br>13. Terpenoid backbone biosynthesis (900)<br>14. Limonene and pinene degradation (903)<br>15. Diterpenoid biosynthesis (904)<br>16. Brassinosteroid biosynthesis (905)<br>17. Carotenoid biosynthesis (906)<br>18. Zeatin biosynthesis (908)<br>19. Sesquiterpenoid biosynthesis (909)<br>20. Phenylpropanoid biosynthesis (940)<br>21. Flavonoid biosynthesis (941)<br>22. Anthocyanin biosynthesis (942)<br>23. Isoflavonoid biosynthesis (943)	Biosynthesis of Secondary Metabolites	

24. Flavone and flavonol biosynthesis (944)<br>25. Stilbenoid, diarylheptanoid, and gingerol biosynthesis (945)		
26. Isoquinoline alkaloid biosynthesis (950)<br>27. Tropane, piperidine, and pyridine alkaloid biosynthesis (960)<br>28. Betalain biosynthesis (965)<br>29. Insect hormone biosynthesis (981)		PATRIC
30. Oxidative phosphorylation (190)<br>31. Photosynthesis (195)<br>32. Methane metabolism (680)<br>33. Carbon fixation in photosynthetic organisms (710)<br>34. Reductive carboxylate cycle (CO2 fixation) (720)<br>35. Nitrogen metabolism (910)<br>36. Sulphur metabolism (920)	Energy Metabolism	
37. Geraniol degradation (281)<br>38. 1,1,1-Trichloro-2,2-bis(4-chlorophenyl) ethane (DDT) degradation (351)<br>39. gamma-Hexachlorocyclohexane degradation (361)<br>40. Benzoate degradation via hydroxylation (362)<br>41. Bisphenol A degradation (363)<br>42. Fluorobenzoate degradation (364)<br>43. Biphenyl degradation (621)<br>44. Toluene and xylene degradation (622)<br>45. 2,4-Dichlorobenzoate degradation (623)<br>46. 1- and 2-Methylnaphthalene degradation (624)<br>47. Tetrachloroethene degradation (625)<br>48. Naphthalene and anthracene degradation (626)<br>49. 1,4-Dichlorobenzene degradation (627)<br>50. Trinitrotoluene degradation (633)<br>51. Ethylbenzene degradation (642)<br>52. Styrene degradation (643)<br>53. Atrazine degradation (791)<br>54. Caprolactam degradation (930)<br>55. Metabolism of xenobiotics by cytochrome P450 (980)<br>56. Drug metabolism - cytochrome P450 (982)<br>57. Drug metabolism - other enzymes (983)	Xenobiotics Biodegradation and Metabolism	
